# Identification of binding residues between periplasmic adapter protein (PAP) and RND efflux pumps explains PAP-pump promiscuity and roles in antimicrobial resistance

**DOI:** 10.1371/journal.ppat.1008101

**Published:** 2019-12-26

**Authors:** Helen E. McNeil, Ilyas Alav, Ricardo Corona Torres, Amanda E. Rossiter, Eve Laycock, Simon Legood, Inderpreet Kaur, Matthew Davies, Matthew Wand, Mark A. Webber, Vassiliy N. Bavro, Jessica M. A. Blair

**Affiliations:** 1 Institute of Microbiology and Infection, College of Medical and Dental Sciences, University of Birmingham, Edgbaston, Birmingham, United Kingdom; 2 School of Life Sciences, University of Essex, Colchester, United Kingdom; 3 Public Health England, National Infection Service, Porton Down, Salisbury, Wiltshire, United Kingdom; 4 Quadram Institute Bioscience, Norwich Research Park, Norwich, United Kingdom; Emory University School of Medicine, UNITED STATES

## Abstract

Active efflux due to tripartite RND efflux pumps is an important mechanism of clinically relevant antibiotic resistance in Gram-negative bacteria. These pumps are also essential for Gram-negative pathogens to cause infection and form biofilms. They consist of an inner membrane RND transporter; a periplasmic adaptor protein (PAP), and an outer membrane channel. The role of PAPs in assembly, and the identities of specific residues involved in PAP-RND binding, remain poorly understood. Using recent high-resolution structures, four 3D sites involved in PAP-RND binding within each PAP protomer were defined that correspond to nine discrete linear binding sequences or “binding boxes” within the PAP sequence. In the important human pathogen *Salmonella enterica*, these binding boxes are conserved within phylogenetically-related PAPs, such as AcrA and AcrE, while differing considerably between divergent PAPs such as MdsA and MdtA, despite overall conservation of the PAP structure. By analysing these binding sequences we created a predictive model of PAP-RND interaction, which suggested the determinants that may allow promiscuity between certain PAPs, but discrimination of others. We corroborated these predictions using direct phenotypic data, confirming that only AcrA and AcrE, but not MdtA or MsdA, can function with the major RND pump AcrB. Furthermore, we provide functional validation of the involvement of the binding boxes by disruptive site-directed mutagenesis. These results directly link sequence conservation within identified PAP binding sites with functional data providing mechanistic explanation for assembly of clinically relevant RND-pumps and explain how *Salmonella* and other pathogens maintain a degree of redundancy in efflux mediated resistance. Overall, our study provides a novel understanding of the molecular determinants driving the RND-PAP recognition by bridging the available structural information with experimental functional validation thus providing the scientific community with a predictive model of pump-contacts that could be exploited in the future for the development of targeted therapeutics and efflux pump inhibitors.

## Author summary

Active efflux due to tripartite efflux pumps is an important mechanism of clinically relevant antibiotic resistance in Gram-negative bacteria. In this study, we have defined for the first time the residue ranges of the PAPs that are involved in the assembly of the major tripartite efflux systems in *Salmonella* and *E*. *coli* based on structural analysis and functional data. The analysis of conservation within these residue ranges allowed prediction of which PAPs that are able to function with the major efflux pump, AcrB. Further direct validation of the critical roles of these residue ranges by site-directed mutagenesis, allowed us to dramatically narrow the PAP-binding surface determinants. Overall, our study provides a novel understanding of the molecular determinants driving the RND-PAP recognition by bridging the available structural information with experimental functional validation thus providing the scientific community with a predictive model of pump-contacts that could be exploited in the future for the development of targeted therapeutics.

## Introduction

The incidence of multidrug resistant (MDR) infections is increasing globally and the need to understand the mechanisms of this resistance is paramount in order to develop novel therapeutics. Efflux pumps are an important mechanism of antibiotic resistance because they are able to pump diverse antimicrobial compounds out of bacterial cells [1]. Of particular relevance to the issue of MDR infections are the tripartite efflux-systems in Gram-negative bacteria, which are composed of an inner membrane pump (typically belonging to the Resistance, Nodulation, Division or RND-family), an outer membrane channel and a periplasmic adaptor protein (PAP) (previously known as the membrane fusion protein) [2]. The tripartite pumps built around the RND family of transporters are the most clinically relevant class and are found in all Gram-negative bacteria with AcrAB-TolC being the principal RND efflux system in *Salmonella*, *Escherichia coli* and other Enterobacteriaceae [3]. It confers intrinsic resistance to multiple, structurally distinct antimicrobials including clinically and veterinary relevant classes such as the β-lactams and quinolones. Over-expression of AcrAB-TolC, or homologous RND efflux pumps in other species, confers MDR and is a common resistance mechanism found in bacterial isolates from humans and animals [4, 5]. RND efflux pumps are also fundamental to the biology of Gram-negative bacteria; for example AcrB and TolC mutants had impaired biofilm formation [6, 7] and attenuated virulence including reduced colonisation of chickens [8, 9]. This multi-faceted role in the biology of Gram-negative bacteria makes these pumps attractive targets for the development of inhibitors.

Genomes of Gram-negative bacteria encode multiple RND-transporters which pair with a number of PAPs forming a variety of efflux systems with different substrate profiles and distinct cellular roles [8, 10–15]. *Salmonella* has five RND transporters (AcrB, AcrD, AcrF, MdtB/C and MdsB) while *E*. *coli* has six and *Pseudomonas aeruginosa* has more than ten. AcrAB is the principal RND system in *E*. *coli* and *Salmonella* as it is highly expressed and has the broadest substrate range. AcrEF has a similar substrate range but is expressed at much lower levels [8, 16]. *Salmonella* has only four PAPs as AcrD is not encoded alongside its own PAP and requires AcrA for function showing that there is some promiscuity between components of RND systems [17]. In addition, we have previously suggested that AcrE can function with AcrB [18] and in *E*. *coli* AcrA has been shown to function with AcrF [19]. In contrast, there are a limited number of outer membrane channels belonging to the outer-membrane factor (OMF) family [20] compared to a seemingly large variety of PAP-transporter pairs. For example, TolC is the cognate channel for many pumps including AcrAB, AcrEF, AcrAD and MdtABC [20]. This is partially because the OMFs are non-specific conduits that are promiscuous at the level of cargo, leaving the selection of the substrate to the transporter. The obvious promiscuity on the level of OMF-PAP has focused the attention of the scientists in the efflux field and left the recognition between the PAP and (RND)-transporter less studied. These interactions however are crucial for assembly of active efflux complexes, and the potential to interchange the PAP used in an efflux system may provide the bacteria with adaptability and plasticity to circumvent antibacterial agents. In addition, the PAPs themselves have previously been highlighted as good targets against which to develop inhibitor molecules [18, 21, 22] making understanding these interactions increasingly important.

Recent advances in structural understanding of tripartite pump organisation, in particular the development of cryo-EM approaches have allowed a glimpse of the complete assembly. Structures, including the engineered *E*. *coli* AcrAB-TolC complex [23]; the structure of disulfide-stabilised AcrAB-TolC [24], as well as the structures of related MacAB-TolC [25] and lower-resolution native-state MexAB-OprM [26] have provided a near-atomic resolution of the interactions within the pumps and elucidated critical aspects of their energetics and pumping mechanisms. These advances now allow dissection of the protein-protein interactions underlying assembly of these pumps, an understanding that may be critical for development of inhibitors of efflux.

**Fig 1:**
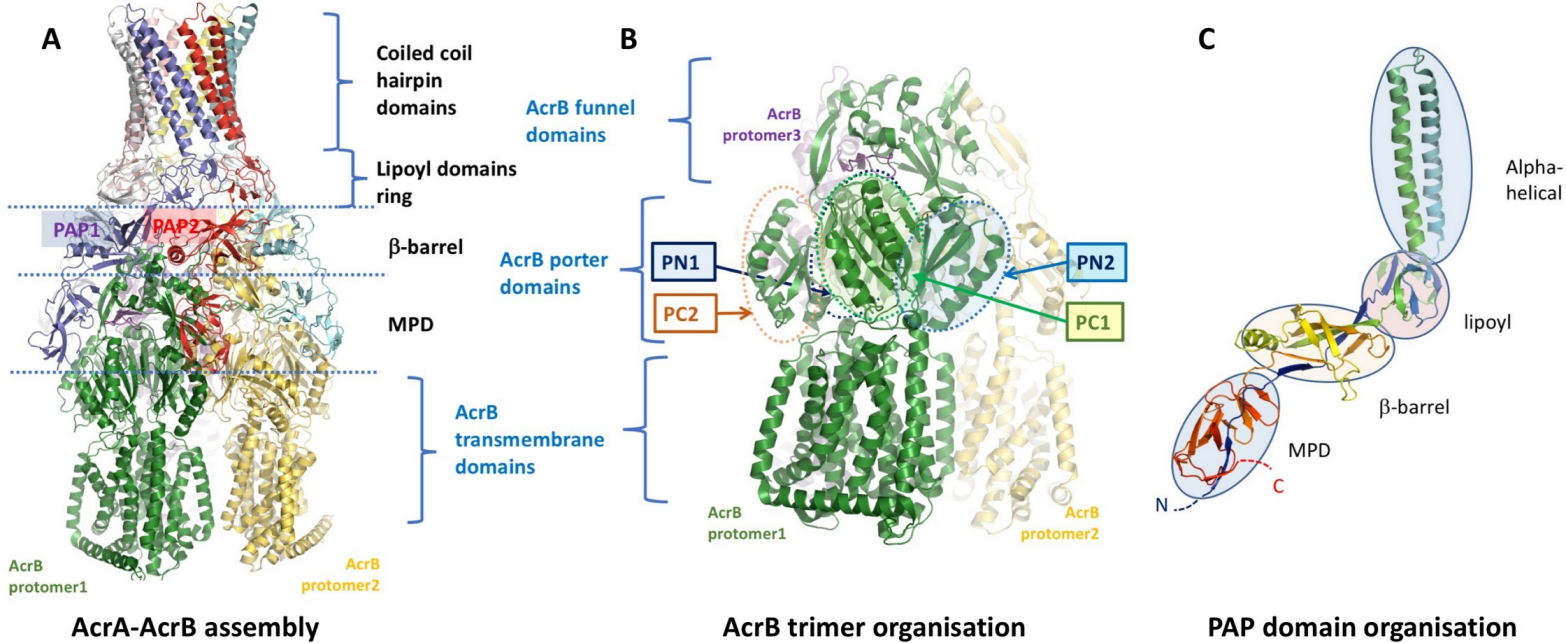
**A.** A general view of the PAP-RND assembly as exemplified by the *E*. *coli* AcrAB sub-complex seen in the asymmetric cryo-EM structure (5o66.pdb) [24]. **B.** AcrB trimer organisation illustrated by side view of the AcrB trimer from the same assembly as in 1A. Protomers coloured with different colours and principal domains indicated. **C.** Domain organisation of a typical PAP based on the experimental structure of *E*.*coli* AcrA (protomer G from 5o66.pdb above). The chain is coloured in rainbow from N-terminus (blue) to C-terminus (red).

**Fig 2:**
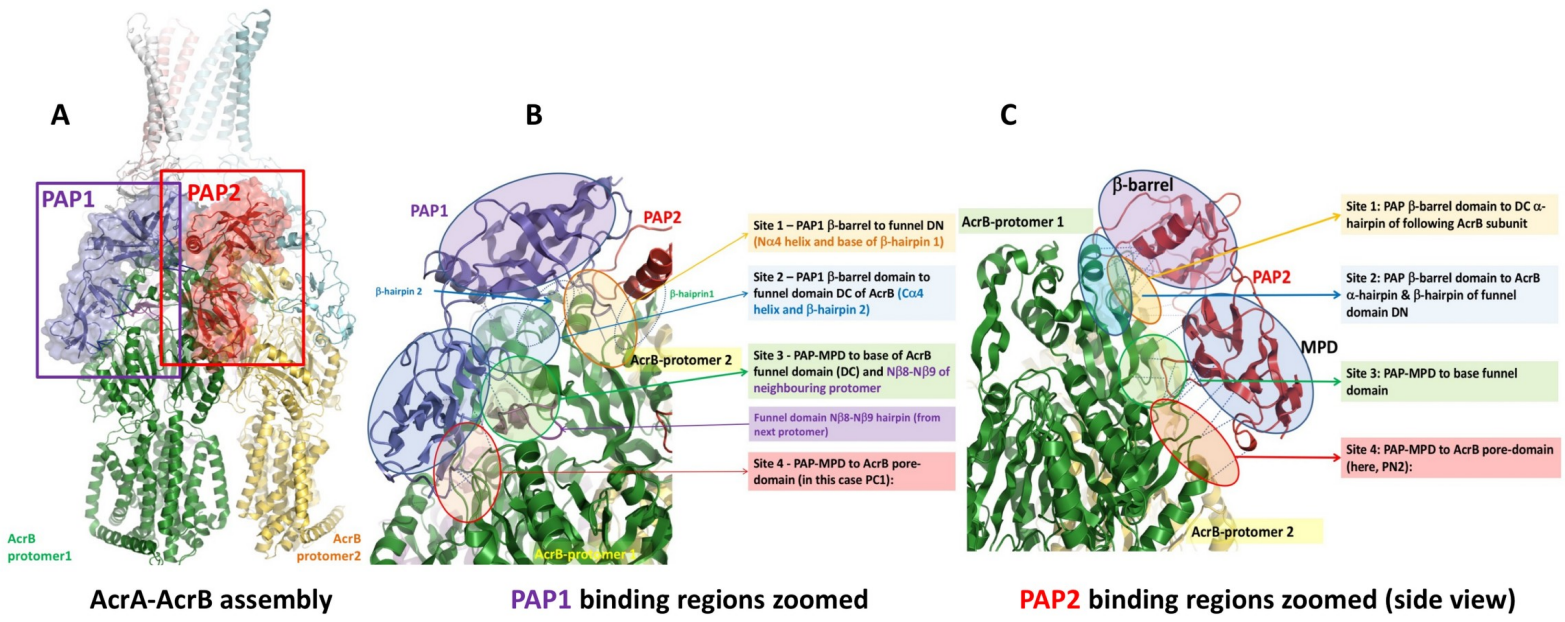
**A.** Positions of PAP1 (violet) and PAP2 (red) protomers relative to RND-protomer within the assembly exemplified by the AcrAB sub-complex discussed in Fig 1 above. Binding is discussed relative to the green protomer of AcrB. **B.** Binding regions of PAP1 relative to the RND protomer (using AcrA chain G in 5o66.pdb for illustration). The region of PAP1-RND interaction is magnified and annotated highlighting the 4 principal binding sites and structural features of the surface. Further details provided in the text. C. PAP2 –RND binding regions on the example of AcrA (chain H) in 5o66.pdb.

The general structure of the RND-efflux pumps as revealed by these data shows an arrangement of 3:6:3 protomers of the OMF, PAP and RND-transporter respectively. The PAP-RND pairs seem to exist in a pre-assembled state, forming a stable binary complex (Fig 1A) [27] that can be isolated and in some cases crystallised as shown for the CusBA metal transporter [28]. The structure of AcrB is shown and described in S1 Fig. The modularity and duplication observed within each RND subunit results in the creation of a pair of semi-equivalent binding sites on the surface of each RND protomer, each supposed to engage with a single PAP although the exact binding mode remains debatable [23, 29, 30]. While in some transporters these two sites are occupied by two separate, dedicated PAPs [e.g. TriABC pump, 31] in the case of AcrAB there are two copies of AcrA per protomer of AcrB which we will refer to as PAP1 and PAP2 (Figs 1A and 2A).

The structures of RND-associated PAPs have been studied in many species [28, 30, 32, 33] although there is no direct structural data available in *Salmonella*. The PAPs have a hairpin-like arrangement with the polypeptide chain beginning at the inner membrane, crossing the periplasm to bridge the outer membrane component of the efflux complex in the middle of the protein and then folding back on itself with the C-terminal being located close to the N-terminal at the inner membrane (Fig 1C) [2]. It is thought that the PAPs provide a link to the OMF (such as TolC) through the α-helical hairpin domain, which has a coiled coil arrangement and may engage with the coiled-coil hairpins of the OMF either by formation of helical bundles [29, 30] or in a tip-to-tip cogwheel-fashion [24, 34] depending on the model of assembly. Adjacent to the hairpin is the lipoyl domain which is composed of four β-strands and is required for interaction of PAPs with each other within the efflux complex [24]. The β -barrel domain is composed of six antiparallel β-strands capped by a single α-helix and is flexibly linked to the membrane proximal (MP) domain. The membrane proximal (MP) domain is formed from the N- and C-terminal regions of the polypeptide and has a beta-roll organisation topologically related to the β -barrel domain [2] (Fig 1C). The MP domain appears to form extensive contacts with the porter-domains of the RND pumps [24] and has been shown to play an important role in substrate acquisition and presentation to the metal-pumping RND transporters [33]. Furthermore in the related ABC-transporter-associated PAPs such as MacA the MP domain appears to be involved in cargo selection and discrimination [35] and activates the ATPase activity of the transporter [36] making this domain a potential target for pump inhibitor design.

Here, we have capitalised on these recent functional insights and combined them with the aforementioned structural biology breakthroughs to determine which PAP residues are involved in the interaction with RND pumps in *Salmonella*. To this end we analysed available docked structures of PAP and RND efflux pumps and showed that the regions of PAP-transporter contact are relatively compact and discrete. Based on homology models of the PAPs in *Salmonella* we found these regions to be highly conserved between AcrA and AcrE but divergent in the other two PAPs—MdtA and MdsA. We furthermore demonstrate that this conservation of binding sites translates into functional promiscuity and redundancy between AcrA and AcrE that manifests in their ability to support efflux function through the major transporter AcrB, while the PAPs lacking conservation in these regions, MdtA and MdsA, cannot. Our findings elucidate residues within PAPs that are important for RND-transporter binding providing a unified framework for future structure-function analysis and also confirm that AcrA and AcrE can function interchangeably which will have implications for the design of efflux inhibitors.

## Results

### Discrete stretches of residues control PAP-RND contact and recognition of the cognate PAP-RND pairs is vetted by a small number of “discriminator” residues

The PAPs have been identified, by us and others, as excellent targets for the development of efflux inhibitors [18, 21, 22] but knowledge of the exact PAP residues important for efflux complex recognition and assembly is limited. The recent near-atomic resolution cryo-EM structures of the stabilized tripartite complex of AcrAB-TolC [24] allow examination of the PAP residues involved in RND-transporter binding. Mapping of the transporter-binding regions derived from the experimental structures reveals several discrete stretches of residues involved in contact. The contacts are provided exclusively from the β-barrel and MP domains (Figs 1A and 2A), while the lipoyl domain is involved in self-association, and the α-helical domains, provide both a contact with the OMF and self-associate to provide a tight seal of the efflux conduit in agreement with the so-called tip-to-tip or cogwheel models of assembly [37].

We analysed the available cryo-EM data, of assembled AcrA-AcrB complexes, to define possible interacting residues. PAPs bind RND transporters in a 2:1 stoichiometry. This results in two protomers of the PAP binding to one protomer of the transporter at different semi-equivalent binding sites with slightly different specificities and affinities [27]. Here we refer to the two PAP positions as PAP 1 and PAP2 (Figs 1A and 2A). Despite the pore domain of AcrB, being composed of the two semi-equivalent lobes (PN1/PC2 and PN2/PC1 respectively) (Fig 2B and in more detail in S1 Fig) our analysis shows that the binding of the AcrA protomers to the surface of the AcrB is strongly asymmetrical, with the binding sites for PAP1 being restricted primarily to the surface of PC1 subdomain of the main protomer to which it is bound, as well as the surface of the funnel-subdomain (Cβ7-Cβ12 hairpin, Cα4 helix and β-hairpin2), with additional strong contribution from the Nβ8-Nβ9 hairpin of the neighbouring AcrB protomer. However, the PAP2 protomer is primarily restricted to the PN2 subdomain of the core AcrB protomer, but also makes contact with the alpha-hairpin of the following AcrB subunit, as well as the funnel domain. The two PAP protomers hence display a significant discrepancy of conformational arrangement, which is expressed primarily in the relative orientation of the MP and β-barrel domains (Figs 2 and S3A). This is further exacerbated by the asymmetry of the AcrB trimers, which results directly from the conformational cycling associated with the pump’s peristaltic function [38, 39]. However, our analysis shows that despite the binding sites on the RND-side being markedly different, essentially the same PAP residues are involved in binding to both, and hence description of the binding sites from the viewpoint of the PAP is easier, as the sites are broadly the same between all 6 PAP protomers, with only some minor deviations. While the exact side-chain orientations may be difficult to deduce due to the medium-to-low resolution of the available structures, they are reliable enough on the level of C-alpha traces, and to define possible binding sites we have considered as “plausible” contacts extending to Cα-Cα distance of 11 Å to account for the level of coordinate uncertainty [40]. Four regions of PAP-contact fulfilling these distance criteria relative to AcrB were defined per AcrB structural repeat, and while some of the contact residues differ depending on the conformation, the key interacting regions remain the same in both PAP protomers. The four discrete “binding sites” in AcrA have been arbitrarily numbered from 1 to 4 (Fig 2B and 2C) and are briefly described below:

**Site 1**: β-barrel domain of PAP 1 to the funnel-domain (DN) of the main RND protomer (alpha-helical fragment Nα4 in Murakami 2002 nomenclature and base of the β-hairpin1) or β-barrel of PAP 2 to the DC domain of the neighbouring RND protomer (α-helical fragment Cα4 and base of β-hairpin2).
**Site 2**: PAP β-barrel domain to β-hairpin of the RND funnel domain (PAP 1- β-hairpin 2 of DC; PAP2 – β-hairpin 1 of DN).
**Site 3:** PAP MP-domain to base of RND funnel domain.
**Site 4**: PAP MP-domain to RND pore (porter)-domain (in the case of PAP1 to PC1; in the case of PAP2 to PN2).

It is striking, that despite the extensive surface area available for a tight interaction between the PAP and the transporter, the two proteins seem to have relatively limited contact, with the main stabilising interactions being restricted to the self-association of the lipoyl domains of the PAPs. This observation by itself directly leads to the suggestion, that from the relatively few remaining contacts, a number will have to be generally preserved for structural rigidity and may be conserved in nature across the PAP-transporter pairs, while a smaller number still will play the role of “discriminators” requiring exact pairing between the PAP and its cognate transporter.

**Fig 3:**
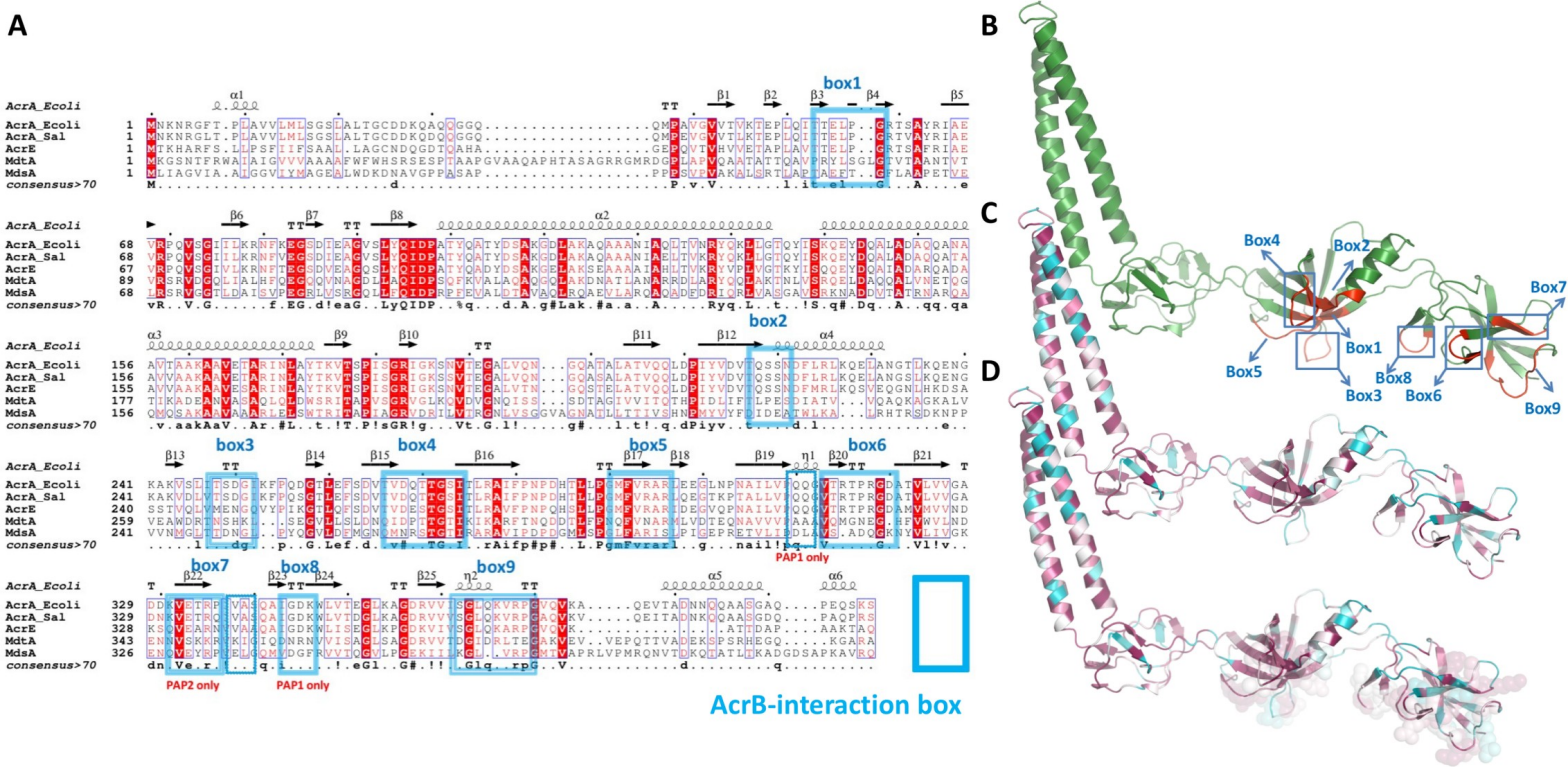
The 3D-binding sites of PAPs relative to the RND-transporter can be reduced to discrete linear sequence “binding boxes”. **A.** A multiple sequence alignment of the 4 *Salmonella* PAPs combined with the structural alignment of the experimental *E*. *coli* AcrA structure (based on 5o66.pdb chainG) (top) reveals clear domain boundaries and correspondingly high likelihood of secondary structure conservation. Identical residues are coloured red. The proposed “binding boxes” are annotated from 1 to 9 and delineated using rectangles. Figure produced using Espript. **B.** Mapping of the *binding boxes* (in blue) onto the 3D structure of the PAPs (AcrA) shows that they are restricted solely to β-barrel and MPD domains and map predominantly to one face of the PAP protomer, which faces the RND transporter. **C.** A Consurf sequence conservation map based on the 150 unique PAP sequences (sequence identity from 95% to 45%) projected onto the AcrA structure. Highly conserved residues are indicated with deep magenta, hypervariable regions in cyan. **D.** A composite image combining the Consurf map from 3C with the space-fill representation of the residues comprising the *binding boxes*, demonstrating the strong conservation of the sequence elements within them.

Taking these considerations into account and to simplify the analysis of the binding sites we have divided them into their actual linear sequence constituents and numbered them from N-terminus to the C-terminus of the PAP (using AcrA as a template). This resulted in 9 discrete “binding boxes” to which we will refer further in the text. They are visualized in the structural alignment (Fig 3A) and mapped onto the structure of the *E*. *coli* AcrA on Fig 3B. A more detailed comparison is provided in S3 Fig.

### Homology modelling of *Salmonella* PAPs reveals two clear structural clusters

To understand more about how these PAP “binding boxes” are conserved amongst the PAP family we conducted further work in *Salmonella*. We have previously reported that the PAPs in *S*. *enterica* display some promiscuity [18] and *Salmonella* is an excellent model that enables study not only of drug resistance phenotypes, but also the effect of efflux on infection. At present there is no direct structural data available for any of the five RND pumps or the four PAPs in the important human pathogen *Salmonella*. Even for *E*. *coli*, structural information for RND-pumps other than AcrAB is not available. Therefore, homology models of *Salmonella* PAPs were designed to allow for structural analysis and sequence conservation mapping. We were able to construct reliable models of *Salmonella* AcrA based on the direct correspondence of the sequence between it and the experimentally determined partial *E*. *coli* structures [32], as well as the full-length cryo-EM structures [24] and the full-length structures of the related MexA from *P*. *aeruginosa* [30].

Based on our analysis, all four *Salmonella* PAPs have the typical four-domain organization of RND-associated PAPs (as in Fig 1C) with clear domain boundaries and produced reliable structural alignments, with correspondingly high-confidence scores of the resulting homology models (S2A Fig). The overall identity between each PAP and the template ranged from 92% for AcrA to below 30% for the MdsA. The protein sequence identity between AcrA and AcrE was 69.3% but structural alignments showed an almost identical secondary structure, with clear domain boundaries and a predicted RMSD of AcrA and AcrE is below 0.5 Å over the full length C-alpha backbone; the corresponding figure for AcrA and MdtA over the core 4 domains is within 0.6 Å (S2B Fig), which is indicative of a very close structural match, although such figures need to account for a possible model bias.

The sequence analysis indicates that the PAPs fall into two subfamilies; while AcrA and AcrE are closely related and form a single phylogenetic branch, both MdtA and MdsA are approximately equidistantly removed from them, with MdsA revealed to be the most divergent amongst the *Salmonella* PAPs (S2A Fig). As a result MdsA was modelled based on the MexA structure as it was the most closely related template with 31.9% identity. A structural overlay of the models of AcrA, AcrE and MdtA is shown in S2B Fig. In particular the overall structure of the β-barrel and MP domain is conserved across the family along with the α-hairpin domain tips which are presumed to interact with the OMF TolC–notably the important ‘RLSD” signature sequence is present in all of them [41] (S2 Fig).

### Conservation analysis of the binding boxes reveals that they are conserved within functional subfamilies but are strongly divergent outside

Comparison of the annotated ‘binding boxes’ in each of the four *Salmonella* PAPs revealed that residues involved in the RND-transporter binding differ between the PAPs subfamilies [42] (Fig 3A and 3B); the binding boxes of AcrA and AcrE are virtually identical while both MdtA and MdsA differ markedly at these sites. Importantly however, the position of these boxes are conserved across the family, have a similar length and are predicted to keep their orientation relative to the transporter in different PAP-RND pairs (S2 Fig). This suggests position is key to function and specific residues define specificity.

We then mapped the conservation of sequence onto the structural model of *Salmonella* AcrA (Fig 3C) and superposed that with the information from the structural analysis of AcrA-AcrB cryo-EM structures that provided the list of interacting residues. The resulting composite (Fig 3D) shows a very strong correlation of conservation in the regions that are contacting the RND transporter within a given family of PAPs. Notably, when we expand the homology search, the residues facing the RND transporters seem to lose their high conservation scores. This is consistent with the evolutionary requirements for preservation of the contacts within a PAP-transporter pair.

In combination with the conservation analysis, the structural mapping of binding interfaces strongly suggests that the discrete “binding boxes” observed above provide the primary mechanism for differentiation between functional PAP-transporter pairs. We therefore reasoned that these sequence differences, should be readily translated into restriction of binding between the different PAPs which could be detected in functional complementation experiments. Specifically, based on conservation of the binding boxes between *Salmonella* AcrA and AcrE we hypothesised that these two PAPs would show promiscuity and interoperability, while the differences observed between AcrA and both MdtA and/or MdsA would preclude their complementation.

### Strains lacking multiple PAPs have reduced efflux, are more susceptible to antimicrobials and have reduced virulence

**Fig 4:**
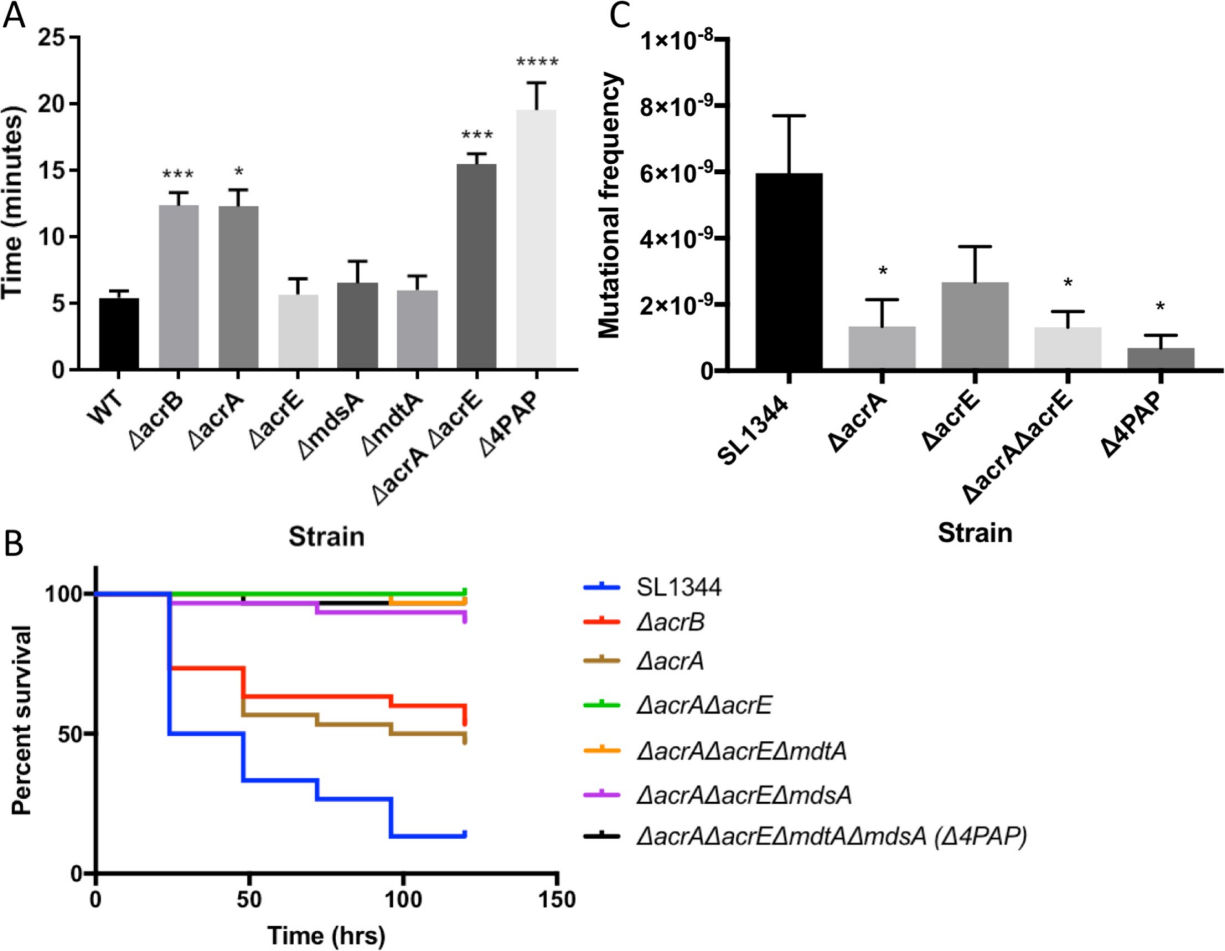
The effect of loss of combinations of PAPs on the phenotype of *Salmonella*. **A**. Efflux of ethidium bromide in strains lacking single or combinations of PAPs. Bacteria were treated with ethidium bromide and CCCP for 60 min and then re-energized with glucose. Data presented is the time taken for the fluorescence to decrease by 25% +/- SE. (Data for 10% and 50% drop can be seen in S1 Table) **B**. Survival of *Galleria mellonella* wax moth larvae infection model. **C**. The frequency of resistance to ciprofloxacin. Data is displayed as the mean of at least 14 biological replicates +/- SE. Data analysed by one-way ANOVA and strains whose frequency of resistance was significantly different (p<0.05) from SL1344 are indicated by *.

In the first instance, to investigate this hypothesis we systematically constructed mutants of *Salmonella* lacking each single PAP and every combination of two, three and four PAPs (Table 1). The only single PAP deletion to alter antimicrobial susceptibility was that of *acrA* while single deletions of *acrE*, *mdtA* or *mdsA* (SE04, SE05 or SE06, respectively) did not alter antimicrobial susceptibility nor effect the rate of efflux of ethidium bromide (Fig 4A). Mutants lacking two or three PAPs only had an altered phenotype if *acrA* was deleted. A strain with intact *acrA* but lacking all three of the other PAPs (*acrE*, *mdtA* and *mdsA*) had the same antimicrobial susceptibility phenotype as the wildtype strain (Table 1) showing that presence of AcrA was sufficient to support normal efflux function in these conditions, presumably through AcrB.

**Table 1:** Antimicrobial susceptibility in strains lacking all combinations of one, two, three or four PAPs.

Strain		Cip	Nal	Chl	Tet	Eryth	Fus	Novo	Oxa	Strep	Acr	CV	EthBr	MB	Rho 6G
WT (SL1344)	SE01	0.015	4	4	2	64	1024	1024	512	8	128	64	1024	1024	1024
*ΔacrB*	SE02	0.008	1	0.5	0.5	4	8	4	4	8	16	4	64	32	8
*ΔacrA*	SE03	0.008	1	1	0.5	2	8	4	8	8	16	4	64	32	16
*ΔacrE*	SE04	0.015	4	4	2	64	1024	512	512	8	128	64	1024	1024	1024
*ΔmdsA*	SE05	0.015	4	4	2	64	1024	512	512	8	128	64	1024	1024	1024
*ΔmdtA*	SE06	0.03	4	4	2	64	1024	512	512	8	128	64	1024	1024	1024
*ΔacrA ΔacrE*	SE07	0.008	1	0.5	0.5	4	4	2	0.5	4	16	2	16	16	8
*ΔacrA ΔmdsA*	SE20	0.008	1	1	0.5	4	8	4	4	8	32	4	64	32	16
*ΔacrA ΔmdtA*	SE21	0.008	1	1	0.5	4	8	4	4	8	32	4	64	32	16
*ΔacrE ΔmdsA*	SE15	0.03	4	4	2	64	1024	512	512	8	128	64	1024	1024	1024
*ΔacrE ΔmdtA*	SE16	0.03	4	4	2	64	1024	512	512	8	128	64	1024	1024	1024
*ΔmdsA ΔmdtA*	SE257	0.015	4	4	2	64	1024	512	512	8	128	64	1024	1024	1024
*ΔacrA ΔacrE ΔmdsA*	SE08	0.008	1	0.5	0.5	4	8	2	0.5	4	16	2	8	16	8
*ΔacrA ΔacrE ΔmdtA*	SE09	0.008	1	0.5	0.5	4	8	2	0.5	4	16	2	32	16	8
*ΔacrA ΔmdsA ΔmdtA*	SE22	0.008	1	0.5	0.5	4	8	4	4	8	16	4	64	32	16
*ΔacrE ΔmdsA ΔmdtA*	SE17	0.015	4	4	2	64	1024	512	512	8	128	64	1024	1024	1024
*ΔacrA ΔacrE ΔmdsA ΔmdtA*	SE10	0.008	1	1	0.5	4	8	2	0.5	4	16	2	16	16	8

Cip, ciprofloxacin; Nal, nalidixic acid; Chl, chloramphenicol; Tet, tetracycline; Amp, ampicillin; Eryth, Erythromycin; Fus, fusidic acid; Novo, novobiocin; Oxa, oxacillin; Strep, streptomycin; Acr, acriflavine; CV, crystal violet; EthBr, ethidium bromide; MB, methylene Blue; Rho 6G, rhodamine 6G.

As shown previously, inactivation of both *acrA* and *acrE* together had an additive effect; an *acrAE* double knockout was significantly more susceptible than either of the single knockouts to ethidium bromide and oxacillin and, to a lesser extent but reproducibly, more susceptible to crystal violet, fusidic acid, methylene blue, norfloxacin, novobiocin and streptomycin (Table 1). This correlated with significantly slower efflux of ethidium bromide (Fig 4A). This suggests that when *acrA* is deleted that AcrE may be partially complementing the mutant phenotype because additional loss of AcrE increased the phenotypic severity in the mutant. No other combination of double PAP deletions had an additive effect compared to the effect of losing only AcrA. Furthermore, deletion of a third PAP from a strain lacking *acrA* and *acrE* or deletion of all four PAPs (*Δ*4PAP) did not further change the phenotype compared to the double *acrA acrE* mutant in terms of antimicrobial susceptibility or efflux rate (Fig 4A and Table 1).

RND efflux provides an intrinsic basal level of resistance to substrate antibiotics. Lack of either *acrB* or *tolC* reduced the frequency with which mutants with decreased susceptibility to substrate antibiotics can be selected but this has not been studied in the absence of the PAPs [43, 44]. Inactivation of the gene coding for the major PAP, *acrA*, significantly reduced the frequency of selection of mutants with deceased susceptibility to ciprofloxacin while inactivation of *acrE* did not. The frequency was also reduced in mutants lacking *acrA* and *acrE* or all four PAPs (*Δ*4PAP, SE10) although the mutant selection frequency was not significantly different from that of the single *acrA* knockout (Fig 4C).

RND efflux is required for virulence in Gram negative bacteria [45]. Deletion of either the major RND pump AcrB or the PAP AcrA significantly increased survival of the *Galleria* wax moth larvae model of infection compared to wild type (53.0% and 46.7% compared to 13.3%, respectively) (Fig 4B). Single deletion of *acrE*, *mdtA* or *mdsA* did not significantly alter Galleria survival compared to WT (S5A Fig). Importantly, deletion of *acrA* and *acrE* had an additive effect causing *Salmonella* to lose the ability to kill the larvae with larval survival increasing to 100%. Strains lacking three or four PAPs were also avirulent in this model. This pattern was confirmed for selected strains in the mouse model of infection. CFU were enumerated from liver and spleen three days after intraperitoneal injection. The number of CFU per liver/spleen was not significantly changed after deletion of only *acrA*, but was significantly reduced upon deletion of *acrA* and *acrE* or deletion of all 4 PAPs (S5B Fig).

RND efflux is also required for biofilm formation so the ability of the mutants to form biofilm was added. None of the PAP mutants tested had a significantly altered ability to form biofilm in our model (S5C Fig).

Together these data support the structural analysis suggesting interoperability of AcrA and AcrE but not MdtA and MdsA because the effect of inactivating *acrA* and *acrE* was additive but this was not true for *mdtA* or *mdsA*.

### Only AcrA or AcrE can complement the *Δ*4PAP mutant phenotype

To investigate the hypothesis that AcrA and AcrE would be interoperable, but that MdtA and MdsA would not, the *Δ*4PAP strain was separately complemented with plasmids encoding one of the four PAPs. Under standard laboratory conditions the major pump AcrB is expressed at much higher levels than any of the other RND pumps and inactivation of the other pumps does not alter antimicrobial susceptibility, so any complementary effect seen following PAP expression seen will be meditated by forming a complex the AcrB pump.

Complementation of the *Δ*4PAP strain with pET20b *acrA* increased MICs of most antimicrobials compared with the *Δ*4PAP strain although not to wildtype levels (Table 2). This is unsurprising as complementation with *acrA* presumably restored function of the major AcrAB-TolC efflux pump. As suggested by the structural predictions, complementation with the secondary PAP, AcrE, was also able to increase the MIC of many of the same antimicrobials and dyes including acriflavine, ethidium bromide, methylene blue, novobiocin and rhodamine 6G although in some cases to lower levels than following complementation with *acrA*. Complementation of the *Δ*4PAP strain with pET20b *mdtA* or pET20b *mdsA* did not alter susceptibility to any of the agents tested suggesting that these two PAPs are not able to form promiscuous interactions with AcrB even when over-produced compared to normal expression level.

**Table 2:** Antimicrobial susceptibility following complementation of the Δ4PAP strain with each individual PAP.

		MIC μg/ml												
Strain	Cip	Nal	Chl	Tet		Acr	Amp	CV	EthBr	Eryth	Fus	MB	Novo	Rho 6G
WT	SE01	0.015	4	4	2	128	2	64	>1024	64	>1024	>1024	1024	>1024
*ΔacrA ΔacrE ΔmdsA ΔmdtA* (*Δ*4PAP)	SE10	<0.008	1	1	0.5	16	0.12	2	16	4	8	16	2	8
*Δ*4PAP *+* pET20b *EV*	SE25	<0.008	1	1	0.5	16	>16	2	16	4	8	16	2	8
*Δ*4PAP *+* pET20b *acrA*	SE26	<0.008	1	2	0.5	64	>16	8	256	32	256	256	32	256
*Δ*4PAP *+* pET20b *acrE*	SE31	<0.008	1	1	0.5	64	>16	8	256	8	32	256	16	64
*Δ*4PAP *+* pET20b *mdsA*	SE32	<0.008	1	1	0.5	16	>16	2	16	4	8	16	2	8
*Δ*4PAP *+* pET20b *mdtA*	SE33	<0.008	1	1	0.5	16	>16	2	16	4	8	16	2	8
*Δ*4PAP *+*pTrc *acrE*	SE12	0.015	4	4	2	128	>16	32	>1024	32	>1024	>1024	1024	>1024

Cip, ciprofloxacin; Nal, nalidixic acid; Chl, chloramphenicol; Tet, tetracycline; Amp, ampicillin; Eryth, Erythromycin; Fus, fusidic acid; Novo, novobiocin; Strep, streptomycin; Acr, acriflavine; CV, crystal violet; EthBr, ethidium bromide; MB, methylene Blue; Rho 6G, rhodamine 6G.

**Fig 5:**
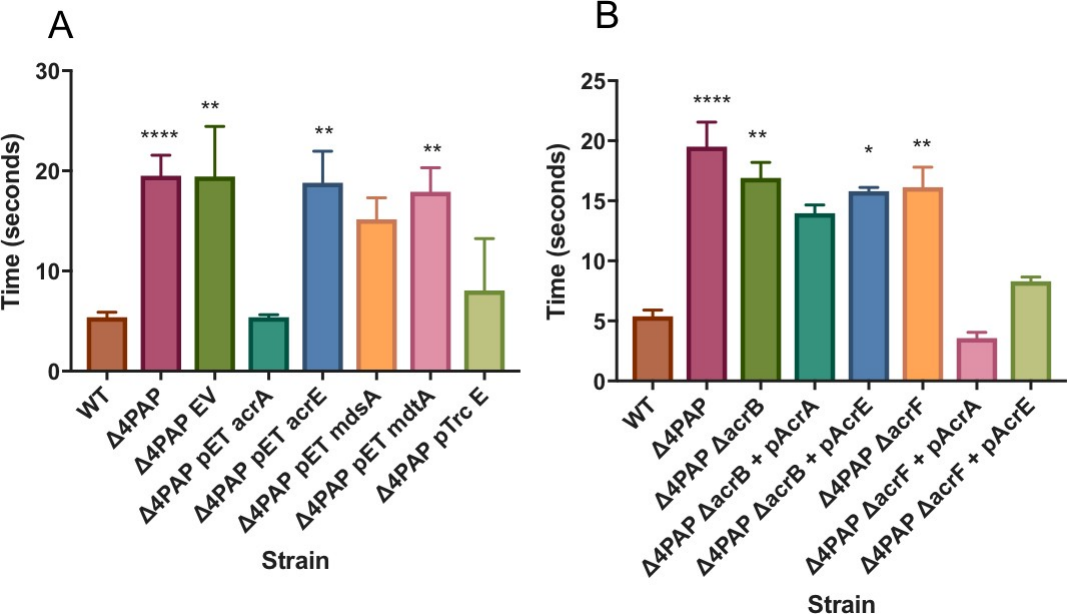
Efflux of ethidium bromide. Bacteria were treated with ethidium bromide and CCCP for 60 min and then re-energized with glucose. Data presented is the time taken for the fluorescence to decrease by 25% +/- SE. (Data for 10% and 50% drop can be seen in S1 Table). A. Shows data for the *Δ*4PAP strain with and without complementation of single PAPs B. Shows data for *Δ*4PAP that also lacks AcrB or AcrF with and without complementation with *acrA* or *acrE*.

One might expect that the effect of expressing *acrE* from the plasmid in the *Δ*4PAP strain should be the be the same as strain SE22 which lacks *acrA*, *mdtA* and *mdsA* but still has its chromosomal copy of *acrE* intact but this is not the case. This is likely because the level of *acrE* produced from the pET20b construct represents over-expression compared to the level produced from the chromosomal copy in wildtype and also in SE22. This shows that the extent of the complementation is therefore heavily dependent on how much of the AcrE protein is present. To investigate the effect of this further *acrE* (and each of the other PAPs) was cloned into a higher copy plasmid (pTRC) and this revealed different patterns. As previously described, very high level over-expression of *acrA* was tolerated poorly by the cell, causing slow growth rate, filamentation and no phenotypic complementation (S1 Table and S6 Fig) [19]. The greater level of AcrE expression was able to complement the antimicrobial susceptibility phenotype of the *Δ*4PAP strain to the same level as the wild type and for a greater range of antimicrobials and dyes including erythromycin, fusidic acid and nalidixic acid and restored efflux of ethidium bromide (Table 2 and Fig 5A). In other words either AcrA or a high level of AcrE, is able to complement the phenotype caused by lack of all four PAPs. However, even when produced at this much higher level, neither MdtA or MdsA provided any complementation of efflux phenotype of the *Δ*4PAP mutant confirming the hypothesis that they are not capable of forming the same promiscuous or redundant interactions as AcrA or AcrE.

### AcrE can function with AcrB

In the strains used for the complementation experiments only the gene coding for the PAP was cloned into the plasmid and over-expressed, not the RND pump. Given that under standard laboratory conditions the major pump AcrB is expressed at much higher levels than its homologue AcrF and that inactivation of *acrE*, *acrF* or *acrEF* does not alter antimicrobial susceptibility, it seemed likely that overexpression of AcrE could be exerting its complementary effect by working with the AcrB pump rather than, or as well as, its native AcrF system. In order to confirm this strains were constructed that lacked all four PAPs and one of the pump genes, *acrB* or *acrF*. These quintuple knockouts were complemented with plasmids encoding one the PAPs, either *acrA* or *acrE*. Deleting the genes coding for either of the pumps AcrB or AcrF in a strain already lacking all 4 PAPs did not have a significant impact on the antimicrobial susceptibility, presumably because the lack of PAPs has already rendered these pumps non-functional (Table 3). Complementation with either *acrA* or *acrE* was not able to increase MICs if AcrB was absent suggesting that it is the AcrB pump that is the major mediator of rescue in the complementation experiments, not AcrF. Crucially, complementation with *acrE* still increased MICs to substrate antibiotics, increased efflux rate and decreased accumulation of ciprofloxacin even in the absence of its cognate pump, AcrF, while in the absence of AcrB it could not (Table 3 and Figs 5B and S6C). This shows that AcrE is exerting its complementary effect by interaction with AcrB as well as, or instead of, AcrF.

**Table 3:** Antimicrobial susceptibility in *Δ*4PAP strain also lacking major pumps AcrB or AcrF and complemented with *acrA*, *acrE*, *mdtA* or *mdsA*.

		MIC μg/ml												
		Nal	Chl	Tet	Amp	Eryth	Fus	Novo	Strep	Acr	CV	EthBr	MB	Rho 6G
WT	SE01	4	4	2	2	64	>1024	1024	8	128	64	>1024	>1024	>1024
*ΔacrA ΔacrE ΔmdsA ΔmdtA (Δ*4PAP)	SE10	1	1	0.5	0.12	4	8	2	4	16	2	16	16	8
*Δ*4PAP *ΔacrF*	SE141	1	1	0.5	0.12	4	8	2	4	16	2	8	8	8
*Δ*4PAP *ΔacrB*	SE143	1	1	0.5	0.12	4	8	1	4	16	2	8	8	8
*Δ*4PAP *ΔacrF +pTrc acrA*	SE168	4	4	1	>16	32	512	256	4	128	16	>1024	>1024	>1024
*Δ*4PAP *ΔacrB +pTrc acrA*	SE176	1	0.5	0.5	>16	4	8	1	4	16	2	8	8	8
*Δ*4PAP *ΔacrF +pTrc acrE*	SE169	4	4	1	>16	32	>1024	512	4	128	32	>1024	>1024	>1024
*Δ*4PAP *ΔacrB +pTrc acrE*	SE177	1	0.5	0.5	>16	4	8	1	4	16	2	8	8	8
*Δ*4PAP *ΔacrF+ pTrc mdsA*	SE170	1	0.5	0.5	>16	2	8	1	4	16	2	8	8	8
*Δ*4PAP *ΔacrB + pTrc mdsA*	SE178	1	0.5	0.5	>16	2	8	1	4	16	2	8	8	8
*Δ*4PAP *ΔacrF+ pTrc mdtA*	SE171	1	0.5	0.5	>16	2	4	1	4	16	2	8	8	8
*Δ*4PAP *ΔacrB + pTrc mdtA*	SE179	1	0.5	0.5	>16	2	8	1	4	16	2	8	8	8
*Δ*4PAP *ΔacrF + pET20b acrA*	SE172	2	2	0.5	>16	32	256	32	4	64	8	128	256	256
*Δ*4PAP *ΔacrB + pET20b acrA*	SE180	2	0.5	0.5	>16	4	8	2	4	32	2	16	8	8
*Δ*4PAP *ΔacrF + pET20b acrE*	SE173	2	1	0.5	>16	8	16	16	4	32	8	128	128	64
*Δ*4PAP *ΔacrB + pET20b acrE*	SE181	1	0.5	0.5	>16	4	8	1	4	16	2	8	8	8
*Δ*4PAP *ΔacrF + pET20b mdsA*	SE174	1	0.5	0.5	>16	4	8	1	4	16	2	8	8	8
*Δ*4PAP *ΔacrB + pET20b mdsA*	SE182	1	0.5	0.5	>16	4	8	1	4	16	2	8	8	8
*Δ*4PAP *ΔacrF + pET20b mdtA*	SE175	1	0.5	0.5	>16	4	8	2	4	16	2	8	8	8
*Δ*4PAP *ΔacrB + pET20b mdtA*	SE183	1	0.5	0.5	>16	4	8	1	4	16	2	8	8	8

Cip, ciprofloxacin; Nal, nalidixic acid; Chl, chloramphenicol; Tet, tetracycline; Amp, ampicillin; Eryth, Erythromycin; Fus, fusidic acid; Novo, novobiocin; Strep, streptomycin; Tric, triclosan; Acr, acriflavine; CV, crystal violet; EthBr, ethidium bromide; MB, methylene Blue; Rho 6G, rhodamine 6G.

### In the absence of AcrA it is possible for select for AcrE over-expression

**Fig 6:**
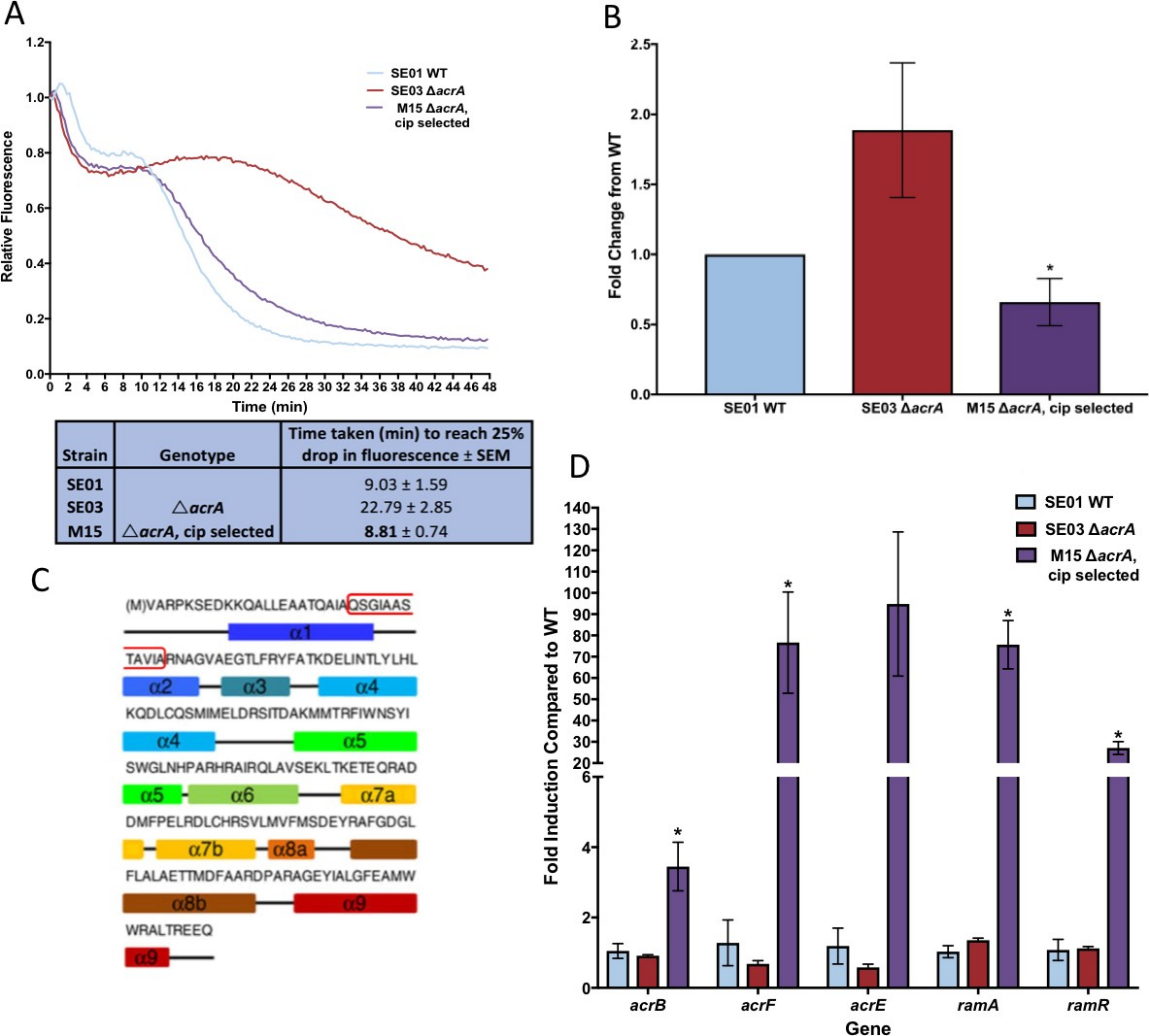
Efflux activity in M15 is restored due to increased expression of *acrEF*. **A.** Ethidium bromide efflux. **B.** Hoechst accumulation. **C.** Schematic of RamR with duplication marked by red box d. Real-time reverse transcriptase PCR. Data from A and B are the mean of three biological replicates +/- SE mean.

The fact that AcrE is able to function with AcrB in the absence of AcrF potentially complicates the issue of finding inhibitors of the PAPs as it suggests that there is potential for resistance to an inhibitor targeted only to AcrA to occur by increased expression of AcrE. In order to see if this is a possibility the mutants with decreased ciprofloxacin susceptibility selected from the *acrA* mutant (Fig 4C) were studied to determine the mechanism of decreased susceptibility. The most common mechanism of resistance to fluoroquinolones is mutations within the quinolone resistance determining region (QRDR) of the *gyrA* gene and more rarely in *gyrB* [46]. The QRDR of *gyrA* was sequenced in some of the selected mutants and selected strains are shown in Table 4. The majority of selected mutants had well described gyrase mutations (e.g. D87G, S83F) explaining their decreased ciprofloxacin susceptibility. However, one mutant, M15, had no *gyrA* or *gyrB* mutations but had increased MICs to ciprofloxacin and other fluoroquinolones and also to ampicillin and erythromycin which are well characterised substrates of RND efflux. In addition, despite lacking *acrA* M15 appeared to have restored efflux as it accumulated similar levels of the Hoechst dye and had similar efflux kinetics as the wild type strain (Fig 6A and 6B). The genome of SE03 (*ΔacrA*) and M15 were sequenced and revealed a 36bp duplication including part of the DNA binding region *ramR* in M15 (Fig 6C). RamR is a TetR family transcription factor that negatively regulates expression of the transcription factor *araC* family regulator RamA, which promotes expression of *acrAB*. RT-PCR revealed that in M15 expression of *ramA* was increased by 76 fold, presumably due to the non-functional RamR protein. In addition, transcription of the gene coding for the secondary PAP *acrE* was increased by 95 fold and its cognate RND pump *acrF* was increased by 77 fold (Fig 6D). Importantly, this shows that in the absence of *acrA*, it was possible to select for a mutant with increased expression of a homologous PAP/pump and that this was sufficient to complement the mutant phenotype back to wildtype levels.

**Table 4:** Antimicrobial susceptibility of ciprofloxacin selected mutants in a *ΔacrA* background and corresponding *gyrA* genotype.

Strain	MIC (μg/ml)				*gyrA* mutation
	Cip	Nal	Amp	Eryth	
SL1344 (WT)	0.03	1	256	4	-
*ΔacrA* (SE3)	<0.008	1	0.25	4	-
M15 (*ΔacrA*)	0.12	16	2	128	None
M16 (*ΔacrA*)	0.015	64	0.25	2	D87G
M17 (*ΔacrA*)	0.03	64	0.25	2	D87G
M18 (*ΔacrA*)	0.06	64	0.25	4	S83F
M19 (*ΔacrA*)	0.03	64	0.25	4	D87G
M20 (*ΔacrA*)	0.06	32	0.25	4	D87G

Cip, ciprofloxacin; Nal, nalidixic acid; Amp, ampicillin; Eryth, Erythromycin.

The very high level over-expression of *ramA* in M15 led to increased expression of *acrB* and both *acrE* and *acrF*. The *acrB* and *acrF* genes were each inactivated in M15 to elucidate whether the efflux restoration detected in this strain was due to high levels of the AcrE/AcrF pair or if promiscuous interactions between AcrE and AcrB were also important for exerting this effect. Inactivation of *acrF* in M15 (Δ*acrA* Δ*acrF*) did not alter the susceptibility to the antimicrobials tested compared to the M15 parent suggesting that the high level of AcrE protein present must be working with the AcrB pump to provide efflux function (Table 5). Inactivation of *acrB* slightly, but reproducibly, reduced MICs to substrate antibiotics. Together this suggests that AcrE/AcrB interactions are the main mediator of the rescued efflux ability in M15 but that AcrE/AcrF complexes are also important.

**Table 5:** Antimicrobial susceptibility of ciprofloxacin selected strain M15 and derivatives.

		MIC μg/ml															
Strain		Fluoroquinolones					Other efflux substrate antibiotics						Dyes				
		Cip	Nal	Levo	Moxi	Nor	Chl	Amp	Eryth	Fus	Novo	Oxa	Acr	CV	EthBr	MB	Rho 6G
WT (SL1344)	SE01	0.015	4	0.06	0.06	0.12	4	2	64	>1024	1024	512	128	64	>1024	>1024	>1024
*ΔacrA*	SE03	<0.008	1	0.03	<0.008	0.03	1	0.12	2	8	4	8	16	4	64	32	16
M15 (*ΔacrA)*	SE186	0.06	8	0.25	0.12	0.5	8	4	128	>1024	1024	1024	256	64	>1024	>1024	>1024
M15 *acrB*::*aph*	SE262	0.03	8	0.25	0.12	0.25	8	2	64	>1024	256	1024	256	32	>1024	>1024	>1024
M15 *acrF*::*aph*	SE263	0.03	8	0.25	0.12	0.25	8	4	128	>1024	512	1024	256	64	>1024	>1024	>1024

Cip, ciprofloxacin; Nal, nalidixic acid; Levo, levofloxacin; Moxi, moxifloxacin; Nor, norfloxacin; Chl, chloramphenicol; Amp, ampicillin; Eryth, Erythromycin; Fus, fusidic acid; Novo, novobiocin; Oxa, oxacillin; Acr, acriflavine; CV, crystal violet; EthBr, ethidium bromide; MB, methylene Blue; Rho 6G, rhodamine 6G.

Together this data validates our structural predictions that AcrA and AcrE can function interchangeably but that MdtA and MdsA do not possess this interoperability. In addition, we have shown that this is biologically relevant because in the absence of AcrA function it is possible to select for compensation by increased expression of AcrE whose binding box sequences were most conserved.

### Site directed mutagenesis validates the critical roles of the binding boxes

**Fig 7:**
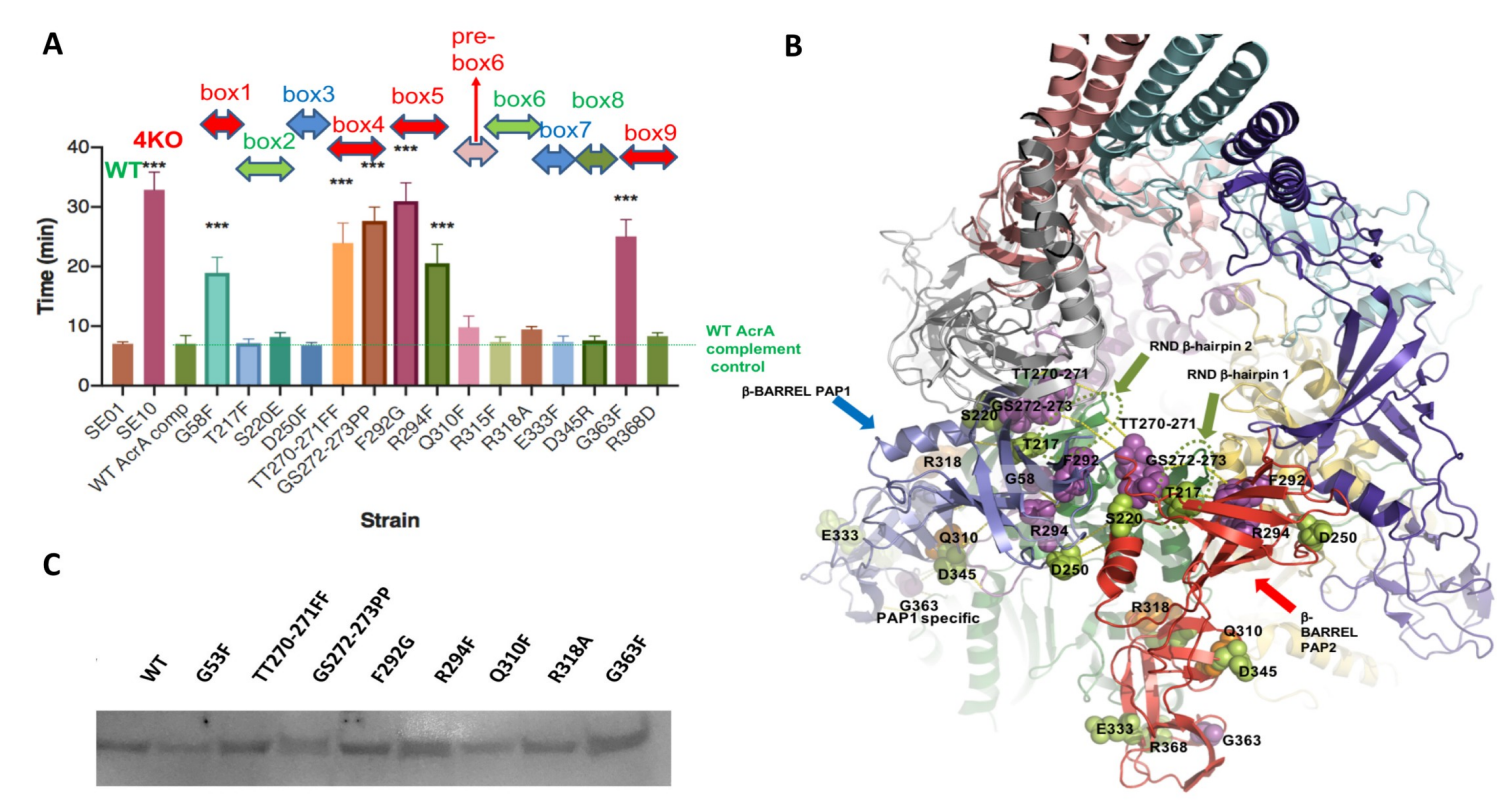
**A.** Efflux of ethidium bromide by strains complemented with mutated versions of AcrA. Mutants labelled above bar according to which binding box is mutated. Bacteria were treated with ethidium bromide and CCCP for 60 min and then re-energized with glucose. Data presented are the mean of three independent biological replicates and are shown as the time taken for the fluorescence to decrease by 50% +/- SE. Data were analysed by one way ANOVA. **B.** Mapping of PAP box mutations to the structure of the assembled complex based on the cryo-EM structure of *E*. *coli* AcrAB-TolC. The PAP 1 protomer bound to the green RND protomer is colored blue; PAP 2 protomer is colored red. For clarity the hairpins of both PAP 1 and PAP 2 protomers are removed. Mutations of residues with prominent phenotypic effect on efflux are colored magenta and the residues responsible are presented in spacefill. Mutations colored orange were responsible for a measurable (although not statistically significant effect) while mutations in green had no measurable effect. The mapping reveals that the majority of the mutations with a statistically significant effect are mapping to the beta-barrel domain of the PAP. In particular, it is notable that the efflux-sensitive mutations cluster around the two beta-hairpins at the crown of the porter domains of the RND-transporter and that the same residues appear to be grasping the hairpin 1 and hairpin 2 respectively in PAP 1 and PAP 2 in a pincer-like fashion. This finding strongly supports the primary role of the beta-hairpins in the PAP assembly. **C.** Western Blot analysis of the expression and stability of selected mutated AcrA constructs with pronounced phenotypic effects. C-terminal His-tagged versions of the proteins were expressed from pET20b in *Salmonella* Δ4PAP background and protein expression visualised using a monoclonal anti-His AP-conjugated antibody.

To further validate the roles of the newly defined sequences boxes we targeted both the most conserved (suggested to form PAP family-wide docking sites) and non-conserved residues (expected to act as discriminators between different PAP-transporter pairs) by site directed mutagenesis followed by quantitative EtBr efflux assay (Fig 7A) and measurement of antimicrobial susceptibility (S2 Table). Mapping of the mutations onto the structure of the AcrABZ-TolC complex [24] is presented in Fig 7B with the mutations with the statistically significant impact on the efflux function coloured magenta. Significantly most of the boxes appear to have a measurable effect on function, with mutations affecting the conserved residues in box 1 (G58F), box 4 (TT270-271FF; GS272-273PP); box 5 (F292G; R294F) and box 9 (G363F) being comparable to the phenotype of the Δ4PAP strain, while measurable impact can also be detected for mutations affecting box 6 (R318A), and intriguingly, mutations in some PAP residues which are predicted to only make contact with the RND transporter in one of the two protomers had a measurably impaired efflux–notably PAP1-specific Q310F (PAP1 specific pre-box 6) (see additional comments in the S1 Text). To validate that the observed effects are not due to changes in protein expression levels or stability of the products we introduced a C-terminal His-tag reporter and quantified protein levels using Western blotting (Fig 7C).

Structural mapping of the mutations revealed that with the exception of the G363 (box 9), the rest of the detrimental mutations belong to β-barrel domain residues of the PAP forming a tight cluster around the β-hairpins (DN and DC respectively) of the AcrB funnel domain, which provides the largest buried surface on the PAP-RND complex. Consistent with this, the mutations within rest of the boxes, that are less conserved and make lower number of interactions with RND protomers had very limited impact on the efflux function.

## Discussion

The RND efflux pumps are an attractive target for inhibition due to their crucial roles in antibiotic resistance, virulence and biofilm formation [e.g. 7, 8, 9, 47]. Several molecules have been found that effectively inhibit RND efflux but for various reasons none of them have progressed to the clinic [48]. One strategy being explored to inhibit efflux is to target the PAP which we previously highlighted [18] and more recently two groups have published studies showing that inhibition of AcrA by small molecules [21] or by antisense technology [22] was indeed sufficient to inhibit efflux.

However, we also suggested that promiscuity existed between AcrA and AcrE in *Salmonella* and that this may have implications for future efflux inhibition strategies [18]. However, until recently reliable structural information about the full-length PAP structure and particularly how it links to the RND transporter has limited our ability to rationalise this finding with structural data and understand how this promiscuity may arise on a molecular level. Recent major advances in the understanding of RND efflux pump structure [23, 24, 26] provided by the advent of high-resolution PAP-RND co-complexes allowed us to identify and systematise the residue ranges involved in PAP-RND binding for the first time. These contact residues form 4 homologous three-dimensional binding sites within each PAP protomer, that translate into 9 discrete linear “*binding boxes”* which are readily identifiable in multiple sequence alignments (Fig 3A). Furthermore, as reported above we demonstrate that PAP residues predicted to be in contact with the surface of the transporter protein are generally well conserved within the related efflux families suggesting evolutionary pressure for preservation of the contacts between the PAP-transporter pair (Fig 3C and 3D). A clear demonstration of such positional residue-conservation linkage can be seen within the MacA PAP family, which form complexes with the unrelated MacB family of ABC transporters. MacA has the same PAP domain architecture as the RND associated ones and importantly utilizes the same structural elements and even residue ranges for binding their cognate transporters although there is very little conservation within the boxes relative to the AcrA-group of PAPs [25].

The observation that there are limited interfaces between the PAP and transporter restricted to a few “*binding boxes*” and the requirement for partner pair-recognition between the PAP and its cognate transporter led us to the straightforward hypothesis of the possible existence of what we called “discriminator residues”. Under this scenario, the limited area of the docking sites, requires the existence and maintenance of robust, and consequently conserved residue pairs, which are responsible for the general docking, while a small subset of residues within the binding boxes, would fulfil the function of recognising the transporter. Closely related PAPs such as AcrA and AcrE, that present correspondingly high conservation within the *binding boxes* are likely to be able to recognise similar transporters. Thus analysis of the boxes can hint at the origin of the promiscuity and functional redundancy and interoperability between the PAPs, while dramatically narrowing the search for the discriminator residues. Consistent with these predictions we have demonstrated that the AcrA and AcrE can functionally complement each other, while the MdtA and MdsA, which function with the significantly divergent RND-transporters MdsB and MdtB/C respectively, fail to do so, again consistent with the high discrepancy of their corresponding binding boxes relative to AcrA.

Crucially, the hypothesis of the role of the conserved residues within boxes being critical for stabilising the structure of the functional tripartite assembly and thus having a measurable effect on the efflux function has been successfully tested using our site-directed mutagenesis (Fig 7 and S2 Table). This revealed that conserved residues belonging to boxes 1, 4 and 5, which create a pseudo-continuous binding site on the surface of the beta-barrel domain are critical for efflux function, which is consistent with our prediction. Notably the same residue ranges in PAP 1 and PAP 2 provide the binding in a pincer-like fashion (Fig 7) around each hairpin, and their apparent tight association is consistent with the primacy of these interactions in maintaining the complex, thus plausibly explaining the impact of the observed mutations. Equally the dramatic effect of mutation of the ultra-conserved G363 residue belonging to box 9 of the MPD demonstrates the importance of these structural anchors. On the other hand, and consistent with the expectations, mutations targeting non-conserved boxes (notably 2 and 3), as well as the PAP-conformer specific boxes such as box 7 and 8, along with the hypervariable residues within box 6 (e.g. R315A) did not have a clearly pronounced phenotypic effect.

While these observations have strongly supported the proposed role of the binding boxes, we furthermore checked the predictive power of our model, by performing an additional “blind” analysis of the MexA from *Pseudomonas aeruginosa*, UniProtKB—P52477 (MEXA_PSEAE) docking mode to MexB based on the structure of the complex which became available after the initial submission of this work [49]. As shown in S7 Fig, (with the location of the tested site-directed mutations in AcrA indicated) despite the evolutionary distance between *Pseudomonas* and *Salmonella*, the structural alignment shows that the boxes align perfectly between the genera and furthermore critically important conserved residues have retained their positions within them.

Our identification of the PAP *binding boxes*, provides a testable hypothesis and a useful framework for further study of PAP-transporter interaction, and while the extensive mutagenesis needed to validate all of them as functional interaction sites goes beyond the remit of the current study, by defining them we were able to provide startling rationalisation of already available data, which lends strong support to this interpretation. For example, previous reports suggest that while *Pseudomonas* PAP MexA isn’t able to interact with TolC, thus rendering the chimeric MexAB-TolC pump inactive [50], the *E*. *coli* AcrA appears to be rather promiscuous and capable of partial interaction with the *Pseudomonas* RND transporter MexB, and that interaction can be further improved by point mutagenesis [51]. Intriguingly, the reported additional AcrA mutations which enabled the TolC-AcrA-MexB pump to gain full function are all located on a continuous stretch of residues from 240–249 in AcrA, coinciding with the position of “*binding box 3”*. The recent cryo-EM structures reveal that the AcrA residues 249–250 are in sufficient vicinity of the RND-transporter to engage in direct contact. Specifically, in 5O66.pdb D732 and K735 and the carbonyl of A803 provide plausible interaction partners from the side of the AcrB to PAP2. This is further reinforced by the observation that one of the MexB adaptive mutations, namely A802V, is located in a position equivalent to the one of A803 in AcrB (as seen in the superposition of 3W9I.pdb [52] with AcrB), suggesting that indeed, the AcrA S249N-MexB A802V reported by Krishnamoorthy et al., presents a correlated mutation pair (S8 Fig) that provides restriction of PAP-RND partners [51]. Thus interaction involving AcrA S249 and AcrB serves as a strong verification tool for the accuracy of the docking of the AcrA-AcrB, and provides a further support for the role of a small number of residues as check-points of assembly or “*discriminators*” vetting the incompatible transporters, and assuring the engagement of the correct cognate ones in agreement with our hypothesis.

Furthermore, previous data on genetic assessment of the role of β-hairpins in the DN and DC domains of AcrB identified compensatory mutations in AcrA, located within binding box 2 (namely S219 *E*. *coli* AcrA full length numbering); and binding box4 (G272;S273) which are revealed by current assembly structures to be in contact with the β-hairpins 1 and 2 of the AcrB DN and DC domains respectively, thus confirming that these binding boxes are directly involved in the pump recognition and assembly [53]. Furthermore, the critical residue G361, mutation of which appears to fatally destabilise the AcrAB-TolC assembly [54], is the key conserved residue within binding box 9 and hence likely plays a crucial structural support role in the recognition process.

Finally, earlier complete pump-reconstruction efforts relying on *in vivo* cross-linking have indicated that only a few residues of AcrA are able to cross-link to AcrB using short-spacer length reactants [30]. It is striking that most of these residues belong to the boxes described–residues 55 (box1); 220 (box2); 250 (box3) from β–barrel domain; residues 320 (box 6); 346 (box8) **and** 376 belonging to the MP domain. Our structural analysis has also provided several additional insights regarding the PAP-RND interaction and these are discussed in the S1 Text.

AcrA and AcrE were virtually identical across the identified binding boxes while MdsA and MdtA, which function with the significantly divergent RND-transporters MdsB and MdtB/C respectively, present a radically different arrangement within the predicted binding sites. This structural data, led to the hypothesis that AcrA and AcrE would be interoperable but that MdtA and MdsA would not. Inactivation of AcrA and AcrE together had an additive effect; compared to loss of just AcrA, efflux activity was reduced, drug susceptibility was increased and virulence decreased suggesting that AcrE is partially complementing the phenotype of the AcrA mutant. However, inactivation, MdtA and/or MdsA, in addition to AcrA and AcrE (Δ4PAP) had no further effect than loss of just AcrA and AcrE and only expression of AcrA or AcrE was able to rescue the mutant phenotype of a Δ4PAP strain while MdtA and MdsA were not. Together this supports the hypothesis that conservation/discrimination based on the predicted binding residues translates into promiscuity or interoperability and redundancy of PAPs/pumps and directly affects the drug susceptibility profile.

The described promiscuity of AcrA and AcrE may explain why we and others have found subtly different phenotypes from inactivation of AcrA and AcrB or both AcrAB [e.g. 22]. This work suggests that when AcrA alone is inactivated, AcrE is partially compensating for its loss. The phenotypic effect of losing AcrB tends to be slightly more severe. There is some reported redundancy between AcrB and AcrF with increased expression of one system to compensate for loss of the other [8, 55]. However, making inactive AcrB protein rather than deleting the gene did not result in this compensatory expression [56]. The regulation of the different efflux systems is complex and it is possible that this compensatory expression is also dependent on other factors.

In addition these data further support the idea that the PAPs could be an effective target against which to develop efflux inhibitors because inactivation of them increased susceptibility to a range of antimicrobials; reduced the frequency at which mutants with other resistance mechanisms could be selected, and reduced virulence. However, we also found that AcrE over-expression could easily be selected for and phenotypically compensate for the loss of AcrA in *Salmonella* and selection for increased expression of homologous efflux systems has also been described in *E*. *coli* [16, 57]. This suggests that inhibition of only a single efflux pump component, for example AcrA, may not be an effective strategy because homologous components can provide PAP function to the major pump AcrB. Our data suggests that, an inhibitor that inhibits at least AcrA and AcrE would provide greater sensitivity to antibiotics, reduction in virulence and prevent resistance to the inhibitor occurring by increased expression of another PAP. However, there was little phenotypic difference between a double AcrAE mutant to a strain lacking all four PAPs so there may be no requirement to inhibit all members of the protein family.

To summarise, in this study we have mapped the residues required for binding of the PAP to the RND pump, identifying critical residues forming discrete *“binding boxes”*. We further validated these by showing that PAPs with conserved *binding boxes* were interoperable while those with a more divergent sequence were not. The discrete nature of the binding sites provides a promising rationale for targeting them with inhibitor molecules and thus decoupling the pumps. This information could also be exploited for creating “designer pumps” with defined engineered characteristics e.g. for improved bioethanol production. Combined with functional *in vitro* analyses, our results suggest a role for PAPs’ β-barrel and MP domains in vetting productive multidrug-efflux complexes. In addition our analysis highlights regions of PAPs critical for the transport mechanism of RND pumps in general; rationalizes previous gain-of-function mutations, and provides the structural basis of PAP-RND recognition, understanding of which will be important for future inhibitor design.

## Materials and methods

### Sequence analysis and modelling the structure of the PAPs

Multiple sequence alignments (MSA) were prepared using MAFFT and NJ/UPGMA phylogeny algorithms as implemented in MAFFT v.7 server (https://mafft.cbrc.jp/).

Structural annotations of the MSA sequences were done with Espript 3 [58]. The AcrA-AcrB interaction surfaces were analysed using InterProSurf [59] as implemented in the Web-server (http://curie.utmb.edu/) using the available cryo-EM structures (5O66.pdb; 5V5S.pdb and 5NIL.pdb) and the results were further cross-validated manually using Coot [60]. Sequence conservation analysis was performed using ConSurf [61]. Additional structural analysis and imaging, including figures was performed with Pymol (PyMOL Molecular Graphics System, Version 1.71 Schrödinger, LLC). For the purposes of homology modelling, we employed I-TASSER [62] in manual mode with assignment of templates and structural alignment.

*Salmonella* AcrA was modelled based on the direct correspondence of the sequence between it and the experimentally determined partial *E*. *coli* structures (2F1M.pdb, residues 53–298) [32] as well as the cryo-EM full-length structures (5O66.pdb chains G and H; 5NG5:E.pdb; 5V5S:D.pdb) [24] and the full-length structures of the related MexA from *P*. *aeruginosa* (2V4D.pdb; chainC) [30]. The templates used for AcrA, AcrE and MdtA were the MP domain-containing AcrA *E*. *coli* structures: 5O66:G.pdb and 5NIL:G.pdb. Due to the lower level of sequence identity, MdsA was modeled using the full-length MexA (2V4D:C.pdb).

### Strain construction and growth

The *acrA*, *acrB and acrE* mutants were constructed previously from *Salmonella enterica* serovar Typhimurium strain SL1344 [9, 18, 47, 63]. Other mutants were constructed using the λ red recombinase system described previously, antibiotic markers were removed and the process repeated to make double, triple, quadruple and quintuple mutants [64]. The PAP genes were amplified by PCR from SL1344 and cloned into pET21b (Novagen), relying upon leaky expression to provide low level complementation of mutant strains. Vectors overexpressing *acrA* or *acrE*, were constructed previously and vectors overexpressing *mdtA* and *mdsA* were generated according to manufacturer's instructions (Invitrogen pTrcHis). Strains were grown in Luria–Bertani (LB) broth at 37°C with shaking unless otherwise stated.

### Antimicrobial susceptibility

The agar doubling dilution method was used to determine the MICs of various antibiotics and dyes according to CLSI guidance. All MICs were repeated at least three times and where necessary a modal value is used. All compounds tested were obtained from Sigma, UK.

### Hoechst accumulation

The efflux activity of the mutants was assessed by determining the accumulation of the fluorescent dye Hoechst H33342 (Sigma, UK) as described previously [65].

### Efflux of ethidium bromide

Efflux activity was also assessed by incubating cells in the presence of ethidium bromide and CCCP. Cells were re-energised and the rate of reduction in fluorescence was measured as previously described [18].

### Ciprofloxacin accumulation

Uptake of ciprofloxacin was measured as previously described [66] with the following adaptations; cultures were grown to an OD_600 nm_ of 0.6, cells were re-suspended in 50 mM potassium phosphate buffer and a viable count was taken. Fluorescence was read using a black microtitre tray in a FLUOstar Optima at excitation and emission wavelengths of 280 and 440 nm, respectively. The fluorescence reading was compared to a standard curve and then divided by the viable count to give amount of ciprofloxacin per cell. Data presented are the mean of three independent biological replicates ±SEM.

#### *Galleria mellonella* killing assays

Wax moth (*G*. *mellonella*) larvae were purchased from Livefood UK Ltd. (Rooks Bridge, Somerset, United Kingdom) and were maintained on wood chips in the dark at 14°C. They were stored for not longer than 2 weeks. Bacterial infection of *G*. *mellonella* was performed essentially as described by Wand et al [67]. Individual *G*. *mellonella* were injected with a bacterial load of approximately 1 x 10^4^ CFU. The data were analyzed by the Mantel-Cox method using Prism software version 6 (GraphPad, San Diego, CA, USA).

### Mouse infection studies

Wild-type BALB/c mice were purchased from HO Harlan Olac Ltd (Bicester, United Kingdom). The mice were maintained under standard animal housing conditions in accordance with local and UK Home Office regulations. Overnight cultures of Salmonella strains for infection studies were inoculated into fresh LB medium at a 1/20 dilution and grown at 37°C to an OD_600_ of 1. The cells from 1 ml of culture were harvested by centrifugation and washed twice with PBS. The cells were resuspended in 1 ml PBS. Female BALB/c mice (8–10 weeks old) were injected intraperitoneally (i.p.) with 3x10^3^ CFU. The exact injected dose was confirmed by plating dilutions of the cell suspension used for infection on LB agar plates. The mice were sacrificed at 3 days post infection and spleens and livers were retained for analyses. To determine the bacterial burden in mouse organs, weighed liver and spleen sections were passed through a 70-μm nylon cell strainer (BD Falcon) with 1 to 5 ml of PBS. The collected cell suspensions were diluted in PBS and plated onto LB agar plates without selection. The recovered colonies were counted and the bacterial burden per whole organ was calculated.

### Biofilm

The ability of mutants to form biofilm was measured using the crystal violet method as described in [7].

### Selection of mutants with decreased susceptibility to fluoroquinolones

Mutant selection experiments were performed with strains SL1344, Δ*acrA*, Δ*acrAE* and *ΔacrAΔacrEΔmdsAΔmdtA* (Δ4PAP) as previously described [44], using 0.06 μg/ml ciprofloxacin for SL1344 and 0.015 μg/ml for all other strains. At least 12 biological replicates were performed for each strain and the mean frequency of resistance was calculated.

### Whole genome sequencing

DNA extraction and WGS was performed by microbes NG, Birmingham. SNPs were identified relative to SL1344 using Snippy v3.2 (https://github.com/tseemann/snippy).

### RNA isolation and RT-PCR

RNA isolation and qRT-PCR were performed as previously described [56] except that the Total RNA Purification Plus Kit (Norgen) was used, cDNA was synthesized from RNA samples using FastGene 55-Scriptase (Nippon genetics) and 16S rRNA was used as a housekeeping gene for data normalisation.

### Site directed mutagenesis

Site directed mutagenesis was performed using QuikChange XL mutagenesis kit (Qiagen) and the StAcrA-pET20b template was used to introduce the mutations. The mutant and WT AcrA proteins were expressed without induction using the leaky background expression in the Δ4PAP derivative of the SL1344 strain as described above.

### Western blotting

For the purposes of expression testing we introduced a C-terminal His-tag (by QuikChange as above) into the StAcrA-pET20b harbouring the mutated versions of the AcrA gene. The constructs were transformed into Δ4PAP derivative of the SL1344 and protein production induced with 0.5mM IPTG at OD_600_ of 0.7. Cells were harvested and lysed in 50mM Tris-HCl 7.5, 200mM NaCl and 10mM β-DDM (Anatrace), supplemented by Completed EDTA-Free proteinases inhibitor tablets (Roche) using Emulsiflex cell disruptor. Cell debris were removed by centrifugation at 20,000g and the supernatant separated using 4–12% Bis-Tris precast gradient SDS-PAGE gel (Invitrogen). Following run the gel was transferred onto PVDF membrane and visualised by anti-6xHis-tag AP-conjugated antibody (Abcam) and visualised using NBT/BCIP chromogenic substrate (Sigma).

## Supporting information

S1 Fig Detailed view of the modular organization of the RND transporters on the example of AcrB. RND transporters, of which AcrB is a prototypical member, are homo-trimeric proteins [68, 69]. In brief, the linear organization of each protomer includes from N- to C-terminus 12 transmembrane (TM) domains, into which (between TM1 and TM2 and between TM7 and TM8 respectively) two large periplasmic loops are spliced. Within each periplasmic loop, there are non-linear arrangements of subdomains–namely in the N-terminal loop a PN1 subdomain, is followed by a split PN2, into which a DN portion of the funnel (or docking) domain is spliced; which is mirrored by the C-terminal loop: PC1 is followed by a split PC2 into which the DC portion of the funnel domain is spliced. To complicate matters further these subdomains then create back-to-front functional pairings, that is–PN1 pairs with PC2 to create one lobe; while PN2 pairs with PC2 to create a second lobe of what is referred to as the porter or pore-domain (Figs 1A and 1B and S1). Furthermore, the funnel domain is organised in a lego-like fashion, with pseudo-continuous beta sheets being formed by the core of the domain’s beta-hairpins (Nβ7-Nβ12 pairing intra-protomer with Cβ8-Cβ9 hairpin) with a contribution of the beta-hairpins from the next/previous protomer (Nβ8-Nβ9 from neighbouring protomer pairing with Cβ7-Cβ12 of the core protomer).
(TIF)

S2 Fig **A.** Side-by-side comparison of the modelled Salmonella PAPs. Due to the lack of reliable structural templates, the N-terminal and C-terminal extensions of MdsA and MdtA (equivalent to E. coli residue ranges 1–37 and 378–397 in the structural alignment) have not been modelled. **B.** Superposed models of the core 4 domains of AcrA (red), AcrE (blue) and MdtA (blue) show the closely matched fold and a predicted RMSD of below 1.3 Å for the entire C-alpha trace.
(TIF)

S3 Fig **A.** Superposition of PAP 1 and PAP2 protomers (on the example of AcrA 5o66.pdb chain G and chain H—in blue and red respectively), demonstrating the discrepancies of relative β-barrel domain and MDP domain orientations. Over the whole chain the RMSD is ~1.3 Å; over the MP domain 0.91 Å; β-barrel domain shows the highest individual discrepancy ~1.16 Å; while α-hairpin and lipoyl domains display 0.55 and 0.66 Å RMSD respectively. **B.** PAP 1 and PAP2 orientation superposed over β-barrel domain and MDP respectively.
(TIF)

S4 Fig Superposition of the modelled *Salmonella* PAPs to illustrate the relative positions of the binding boxes and the discrepancies between AcrA (red), AcrE (green), MdtA (blue) and MdsA (yellow). Superposition done over the C-alphas of the β-barrel and MDP domains respectively. The preservation of the predicted “*binding box*” interfaces relative to the transporter is evident.
(TIF)

S5 Fig The effect of PAP inactivation on **A.** survival of the Galleria wax moth larvae, the ability to **B.** infect the mouse model of infection and C. form biofilm. In the mouse model experiments six animals were infected for each mutant over two independent experiments and the data pooled. Data were analysed using the Mann Whitney test. The biofilm data is shown as the mean of three independent biological replicates +/- standard error.
(TIF)

S6 Fig A. Efflux of ethidium bromide. Bacteria were treated with ethidium bromide and CCCP for 60 min and then re-energized with glucose. Data presented is the time taken for the fluorescence to decrease by 25% +/- SE. When produced on pTrc acrA, mdsA and mdtA do not complement the mutant phenotype. B. Gram stains showing filamentation of the Δ4PAP strain when acrA is overproduced at a high level but not when acrE is produced at a similar level. C. Intracellular accumulation of ciprofloxacin. Data is displayed as the mean of at least three biological replicates each in technical duplicate + SE mean. * denotes a strain with a significantly different accumulation of ciprofloxacin compared to SL1344 (p<0.05 following a two way ANOVA with Dunnett’s multiple comparison test).
(TIF)

S7 Fig Structural alignment of MexA from *Pseudomonas aeruginosa* (top) vs the *Salmonella* and *E*. *coli* PAPs discussed before. The secondary structural elements and the corresponding sequence numbering are based on the structure 2V4D.pdb (UniProtKB P52477). The relative positions of the residues belonging to the binding boxes mutated in the *Salmonella* AcrA and their effect are represented by star signs and colours. The alignment demonstrates that despite the divergent nature of the MexA and the presence of deletions in the sequence (e.g. at positions 117 and 135 corresponding to the hairpin domain) the overall positions and sizes of the boxes are identical, and furthermore, the positions of residues with pronounced effect on efflux are coinciding with the highly conserved residues within the boxes.
(TIF)

S8 Fig PAP1 vs PAP2 in contact with the transporter showing residues of importance rationalising the results reported in Krishnamoorthy et al., 2008. (TIF)

S9 Fig Mapping of the previously identified RND region involved in discrimination of cognate PAP–corresponding to AcrB 60–612 in cyan [50] suggests that the DN/PN2 domains are primarily responsible for the recognition of the PAP from the side of the transporter and correspondingly that PAP2 is primarily involved in transporter recognition, while PAP1 position may be more promiscuous. (TIF)

S10 Fig **A:** comparison of the PAP1 and PAP2 assembling on the CusA surface (left) and AcrB surface (right). Note the clear difference in PAP2 interaction with PC1 and PN2 domains. The lipoyl domains in CusBA complex also are much more vertically extended and present a steeper angle relative to the funnel domain of the transporter. **B:** Superposition of the PAP2 orientation in AcrAB complex (red) and CusBA complex–yellow. For clarity the beta-hairpin domain is removed. While beta-barrel domains are in similar orientation the linker to the MP domain seems to have undergone a large conformational change as a result of which the MP domain is interacting primarily with PC1 domain, but appears to have lost the PN2-domain interactions. Notably the N- and C-termini of the PAP2 in CusBA complex form extended contacts with the cleft between PC1/PC2 subdomains, which is homologous to the entry 2 tunnel of the drug efflux transporters.
(TIF)

S1 Table Complete strain list with generation time and ethidium bromide efflux where measured. Data presented is the mean of at least three independent biological replicates +/- SEM.
(DOCX)

S2 Table Antimicrobial susceptibility of strains complemented with mutated versions of AcrA. (DOCX)

S1 Text Supplementary results and discussion text. (DOCX)
