# Supplementary figures and images for: Identification of binding residues between periplasmic adapter protein (PAP) and RND efflux pumps explains PAP-pump promiscuity and roles in antimicrobial resistance
Source: PLoS Pathog. 2019 Dec 26;15(12):e1008101. doi: 10.1371/journal.ppat.1008101 (PMC6975555; doi:10.1371/journal.ppat.1008101)

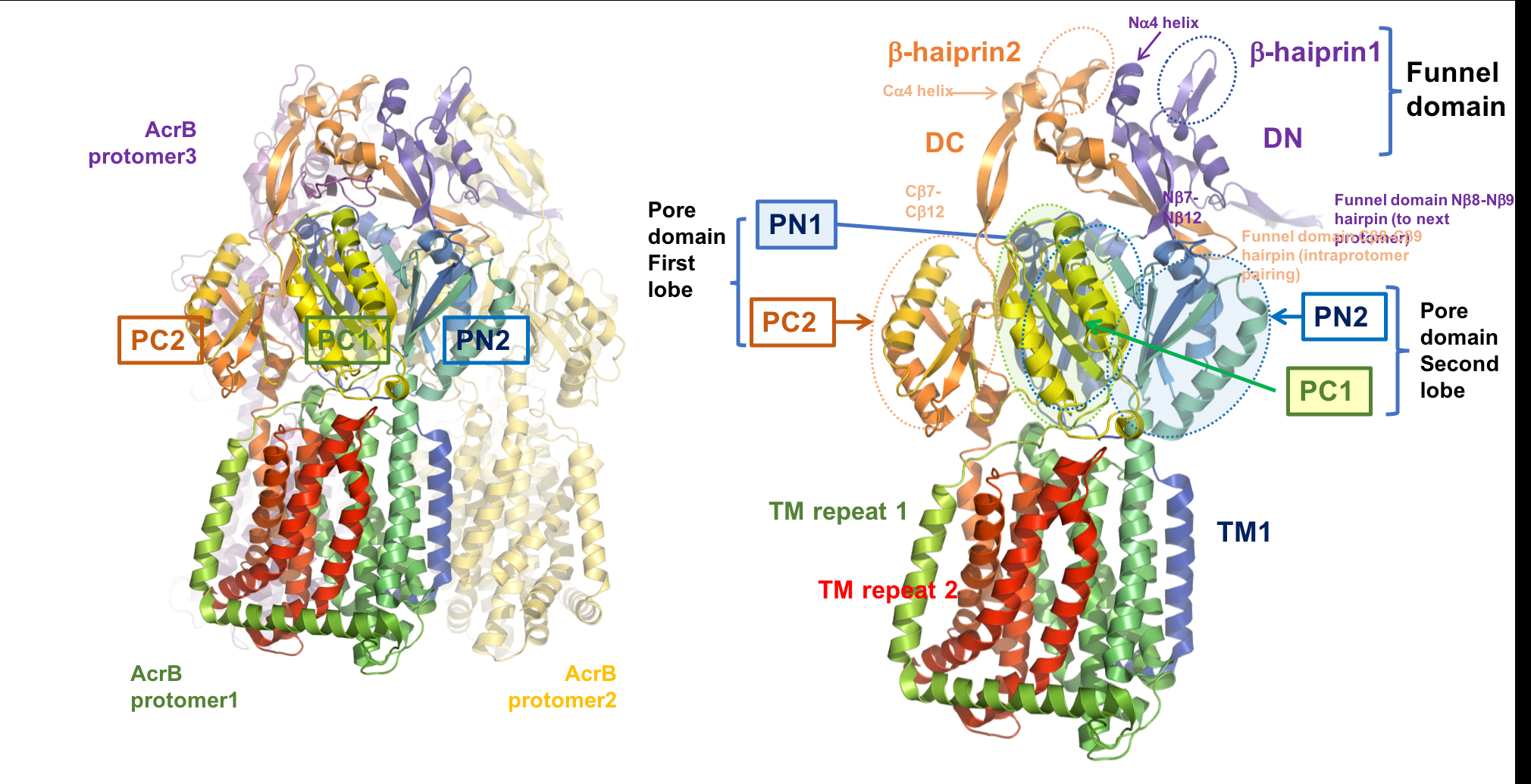

Supplement: S1 Fig — RND transporters, of which AcrB is a prototypical member, are homo-trimeric proteins [68, 69]. In brief, the linear organization of each protomer includes from N- to C-terminus 12 transmembrane (TM) domains, into which (between TM1 and TM2 and between TM7 and TM8 respectively) two large periplasmic loops are spliced. Within each periplasmic loop, there are non-linear arrangements of subdomains–namely in the N-terminal loop a PN1 subdomain, is followed by a split PN2, into which a DN portion of the funnel (or docking) domain is spliced; which is mirrored by the C-terminal loop: PC1 is followed by a split PC2 into which the DC portion of the funnel domain is spliced. To complicate matters further these subdomains then create back-to-front functional pairings, that is–PN1 pairs with PC2 to create one lobe; while PN2 pairs with PC2 to create a second lobe of what is referred to as the porter or pore-domain (Figs 1A and 1B and S1). Furthermore, the funnel domain is organised in a lego-like fashion, with pseudo-continuous beta sheets being formed by the core of the domain’s beta-hairpins (Nβ7-Nβ12 pairing intra-protomer with Cβ8-Cβ9 hairpin) with a contribution of the beta-hairpins from the next/previous protomer (Nβ8-Nβ9 from neighbouring protomer pairing with Cβ7-Cβ12 of the core protomer). (TIF) [file ppat.1008101.s001.tif]

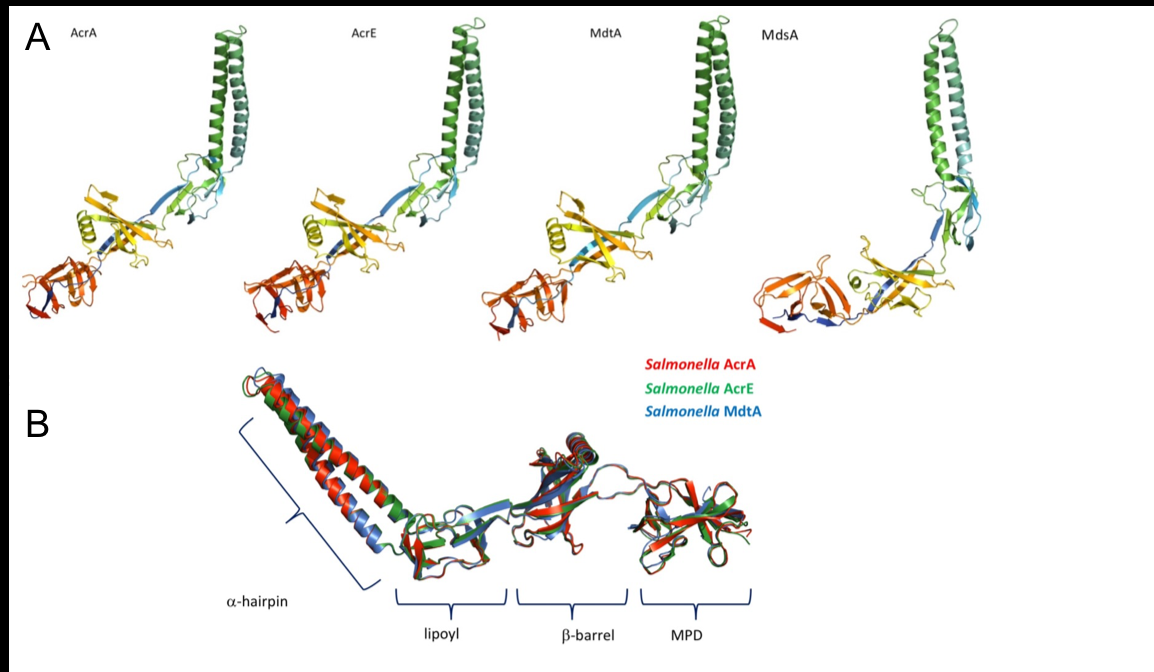

Supplement: S2 Fig — A. Side-by-side comparison of the modelled Salmonella PAPs. Due to the lack of reliable structural templates, the N-terminal and C-terminal extensions of MdsA and MdtA (equivalent to E. coli residue ranges 1–37 and 378–397 in the structural alignment) have not been modelled. B. Superposed models of the core 4 domains of AcrA (red), AcrE (blue) and MdtA (blue) show the closely matched fold and a predicted RMSD of below 1.3 Å for the entire C-alpha trace. (TIF) [file ppat.1008101.s002.tif]

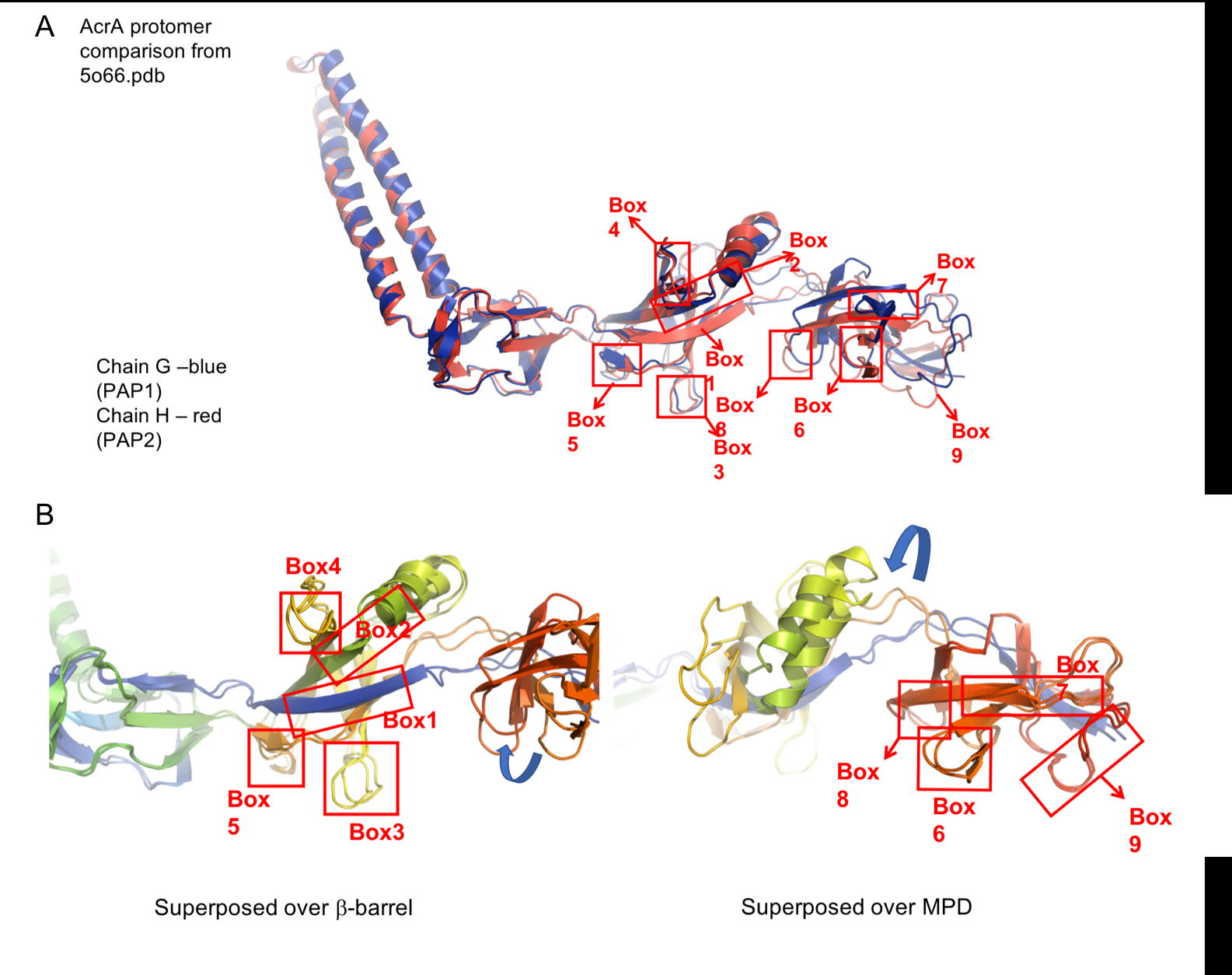

Supplement: S3 Fig — A. Superposition of PAP 1 and PAP2 protomers (on the example of AcrA 5o66.pdb chain G and chain H—in blue and red respectively), demonstrating the discrepancies of relative β-barrel domain and MDP domain orientations. Over the whole chain the RMSD is ~1.3 Å; over the MP domain 0.91 Å; β-barrel domain shows the highest individual discrepancy ~1.16 Å; while α-hairpin and lipoyl domains display 0.55 and 0.66 Å RMSD respectively. B. PAP 1 and PAP2 orientation superposed over β-barrel domain and MDP respectively. (TIF) [file ppat.1008101.s003.tif]

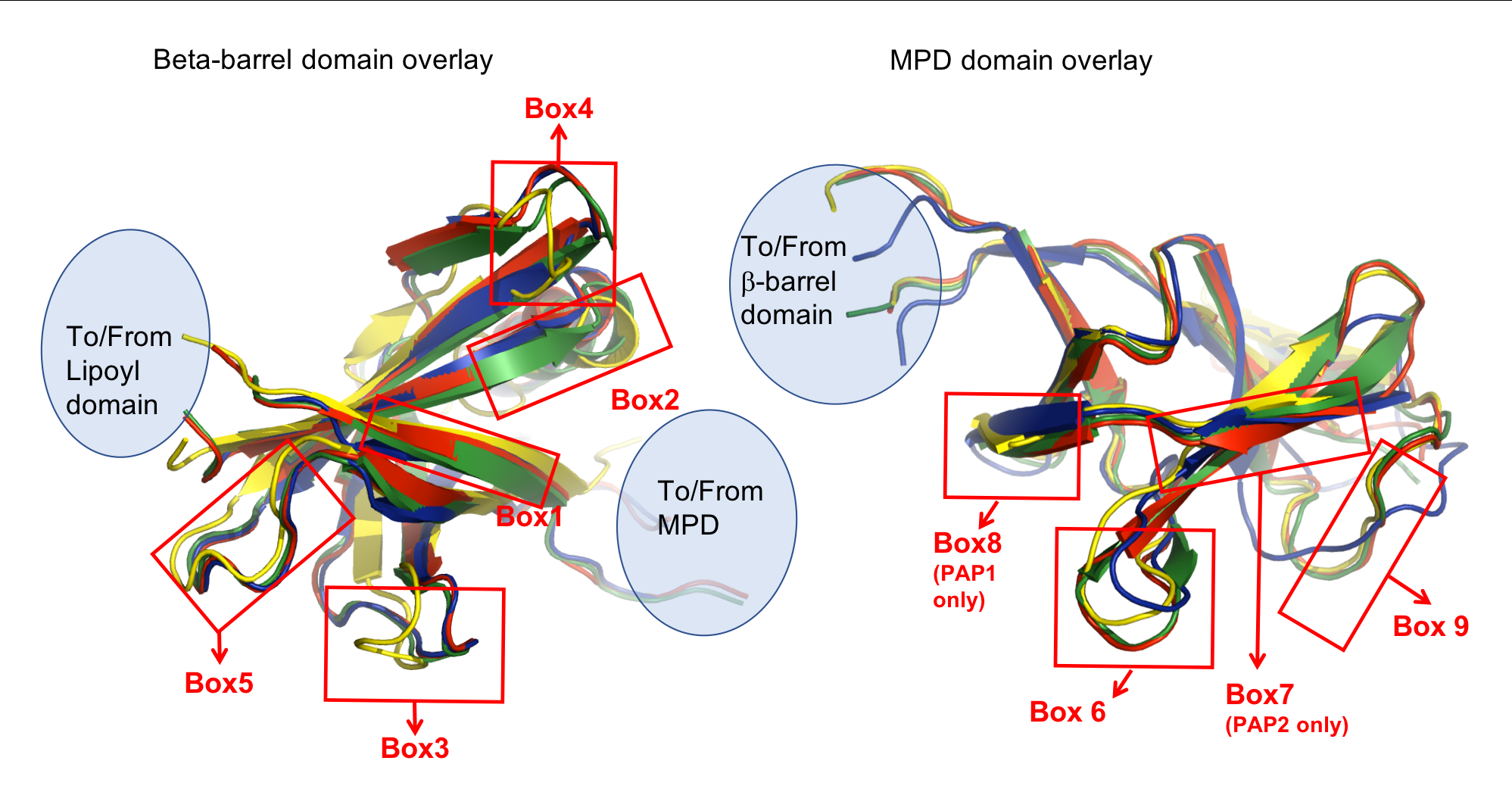

Supplement: S4 Fig — Superposition done over the C-alphas of the β-barrel and MDP domains respectively. The preservation of the predicted “binding box” interfaces relative to the transporter is evident. (TIF) [file ppat.1008101.s004.tif]

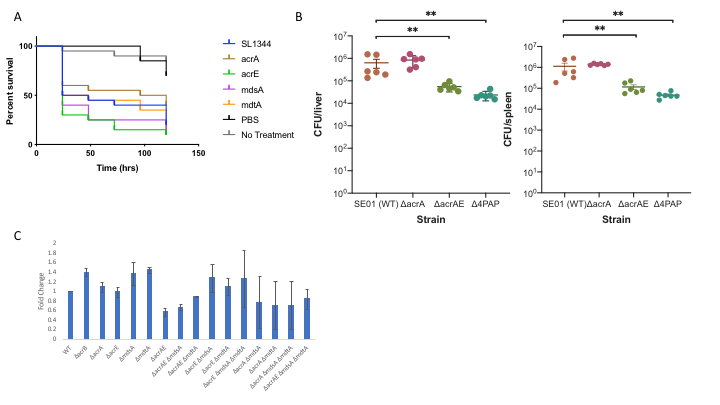

Supplement: S5 Fig — The effect of PAP inactivation on A. survival of the Galleria wax moth larvae, the ability to B. infect the mouse model of infection and C. form biofilm. In the mouse model experiments six animals were infected for each mutant over two independent experiments and the data pooled. Data were analysed using the Mann Whitney test. The biofilm data is shown as the mean of three independent biological replicates +/- standard error. (TIF) [file ppat.1008101.s005.tif]

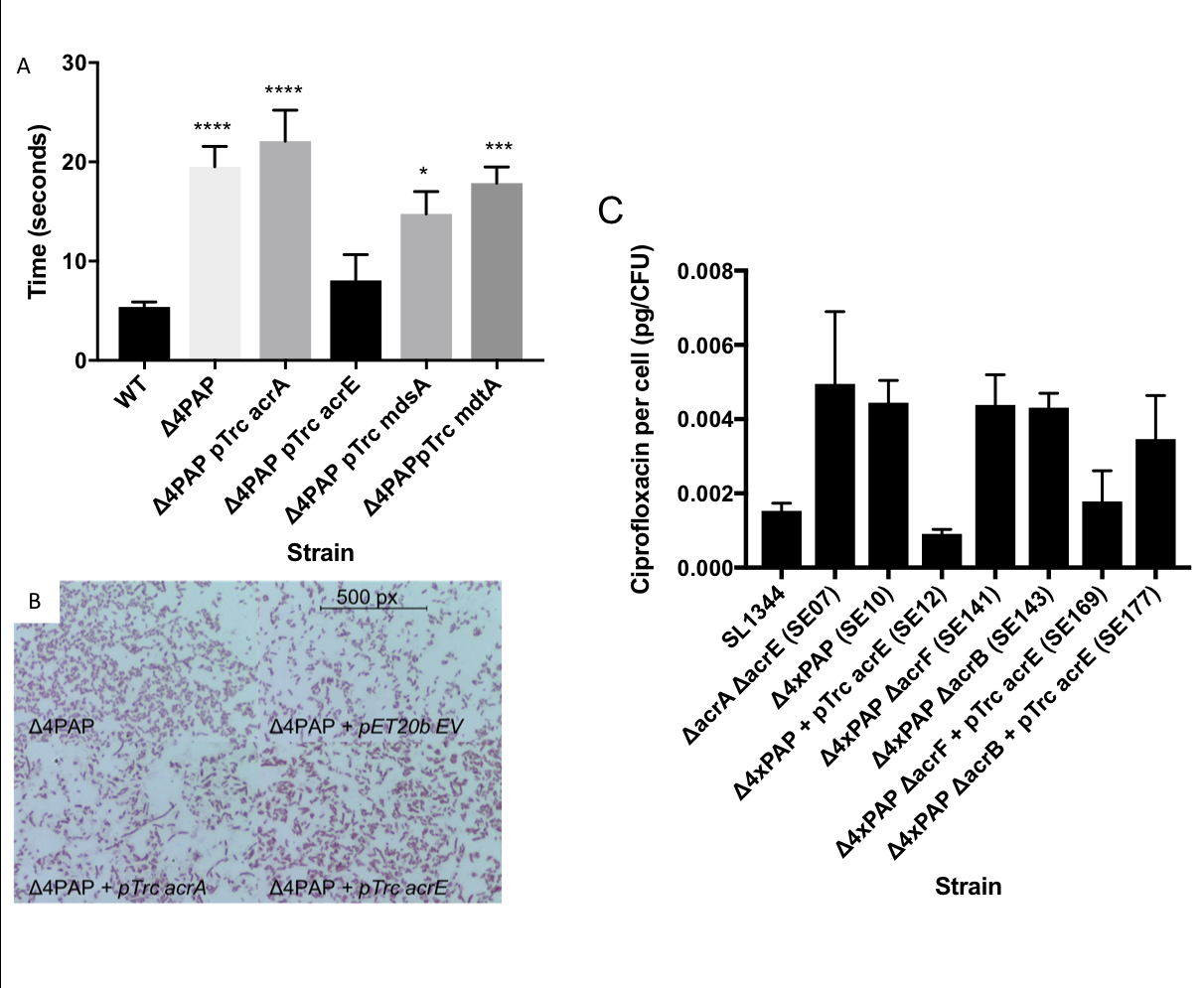

Supplement: S6 Fig — A. Efflux of ethidium bromide. Bacteria were treated with ethidium bromide and CCCP for 60 min and then re-energized with glucose. Data presented is the time taken for the fluorescence to decrease by 25% +/- SE. When produced on pTrc acrA, mdsA and mdtA do not complement the mutant phenotype. B. Gram stains showing filamentation of the Δ4PAP strain when acrA is overproduced at a high level but not when acrE is produced at a similar level. C. Intracellular accumulation of ciprofloxacin. Data is displayed as the mean of at least three biological replicates each in technical duplicate + SE mean. * denotes a strain with a significantly different accumulation of ciprofloxacin compared to SL1344 (p<0.05 following a two way ANOVA with Dunnett’s multiple comparison test). (TIF) [file ppat.1008101.s006.tif]

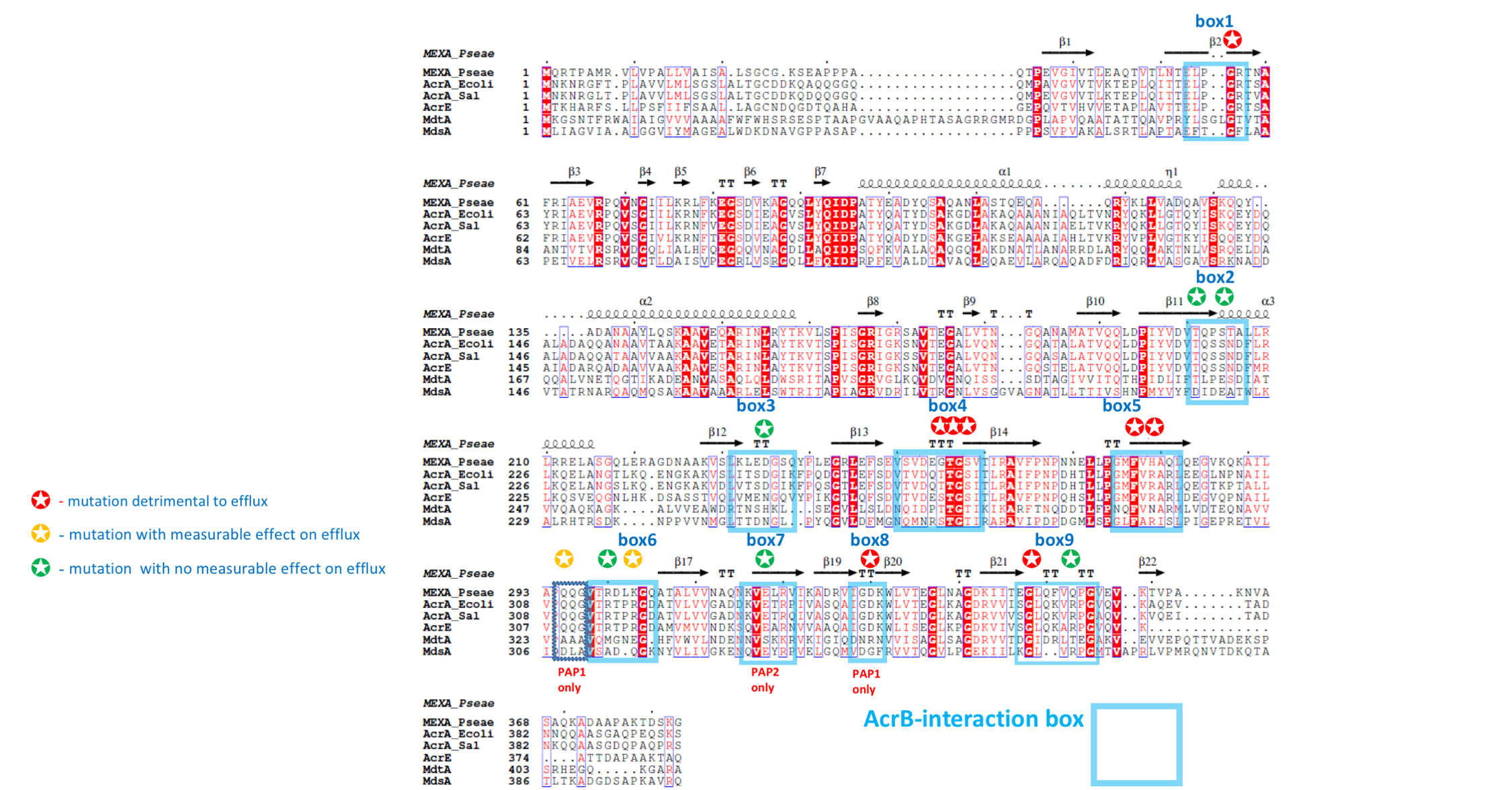

Supplement: S7 Fig — The secondary structural elements and the corresponding sequence numbering are based on the structure 2V4D.pdb (UniProtKB P52477). The relative positions of the residues belonging to the binding boxes mutated in the Salmonella AcrA and their effect are represented by star signs and colours. The alignment demonstrates that despite the divergent nature of the MexA and the presence of deletions in the sequence (e.g. at positions 117 and 135 corresponding to the hairpin domain) the overall positions and sizes of the boxes are identical, and furthermore, the positions of residues with pronounced effect on efflux are coinciding with the highly conserved residues within the boxes. (TIF) [file ppat.1008101.s007.tif]

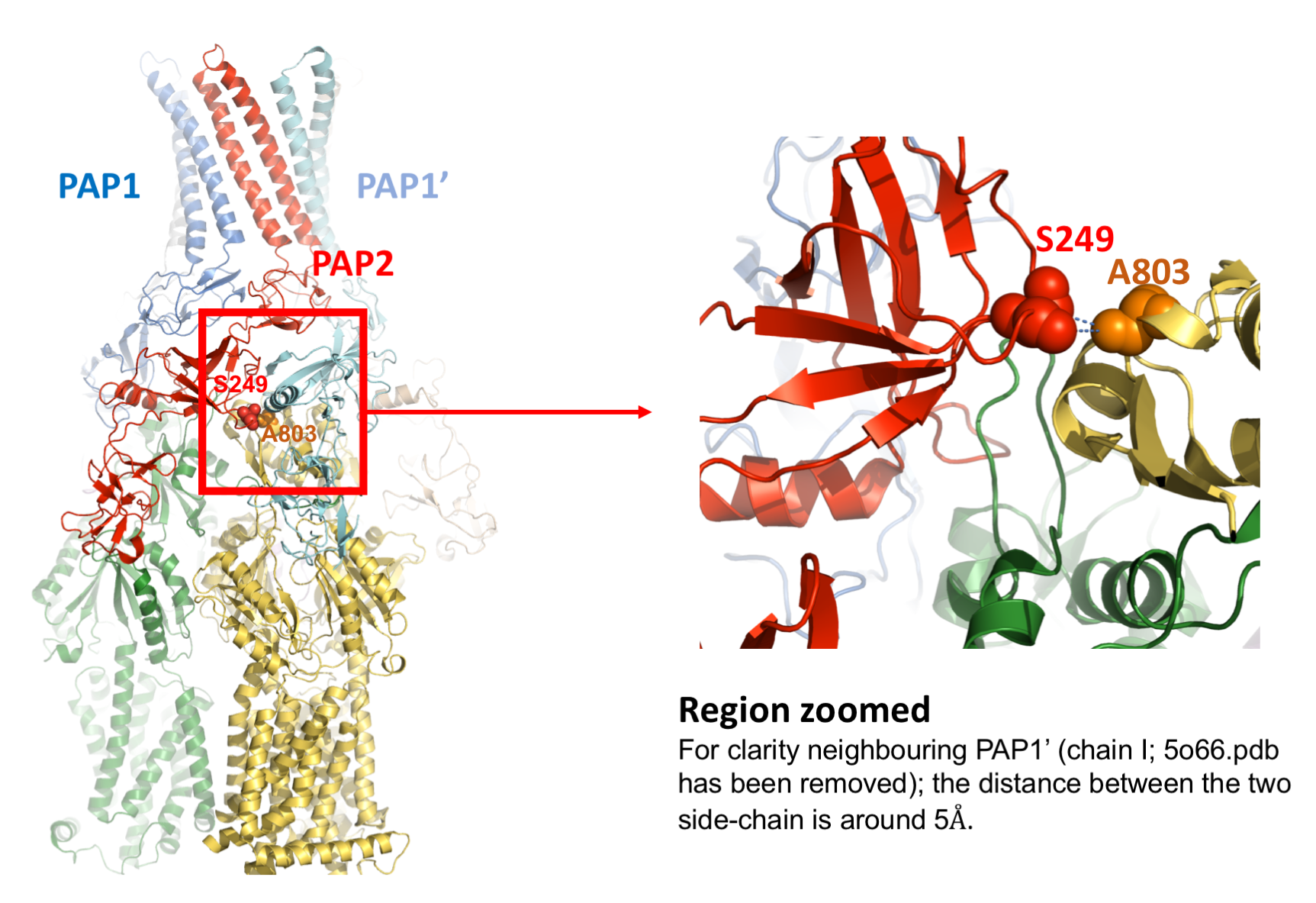

Supplement: S8 Fig — (TIF) [file ppat.1008101.s008.tif]

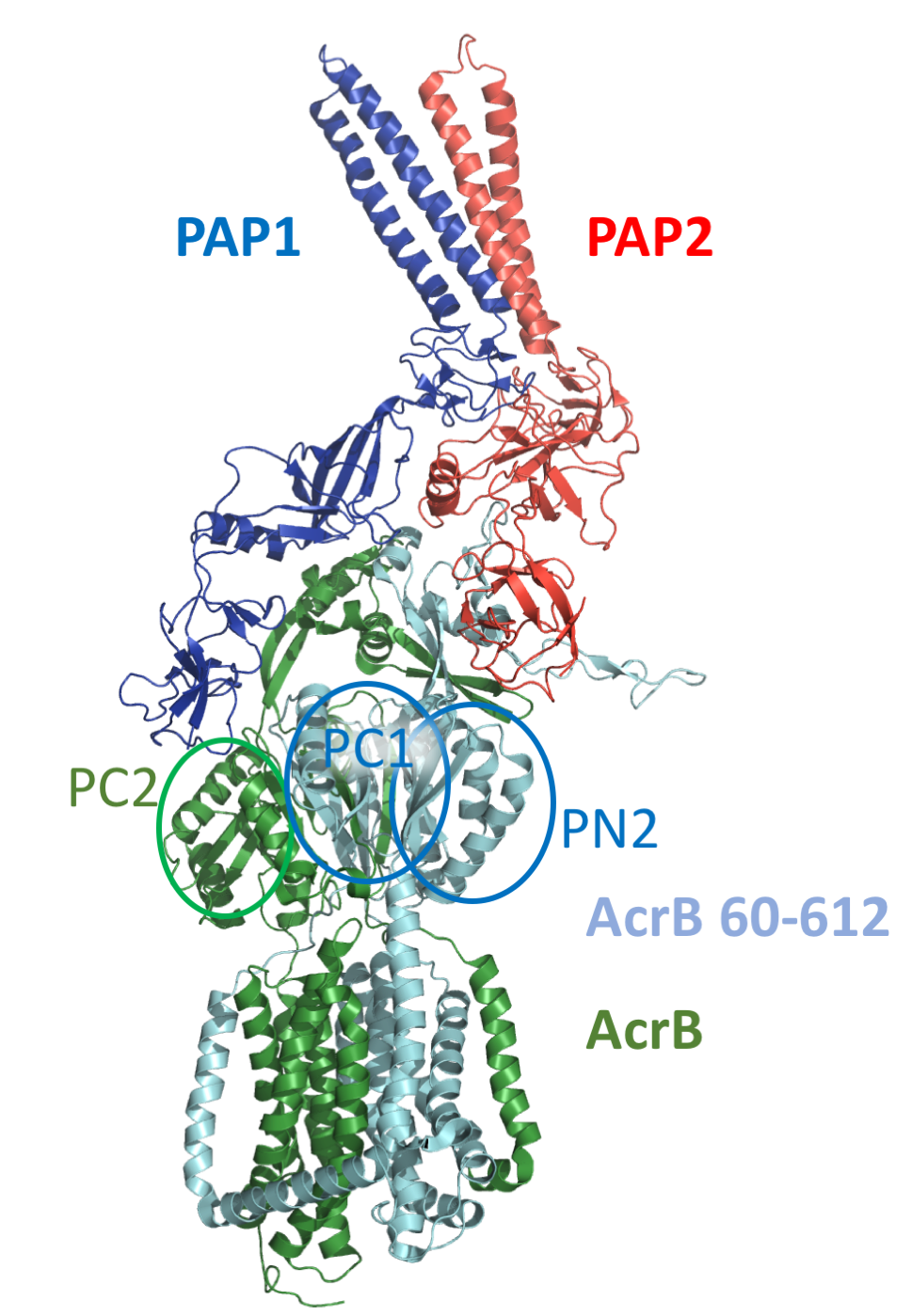

Supplement: S9 Fig — (TIF) [file ppat.1008101.s009.tif]

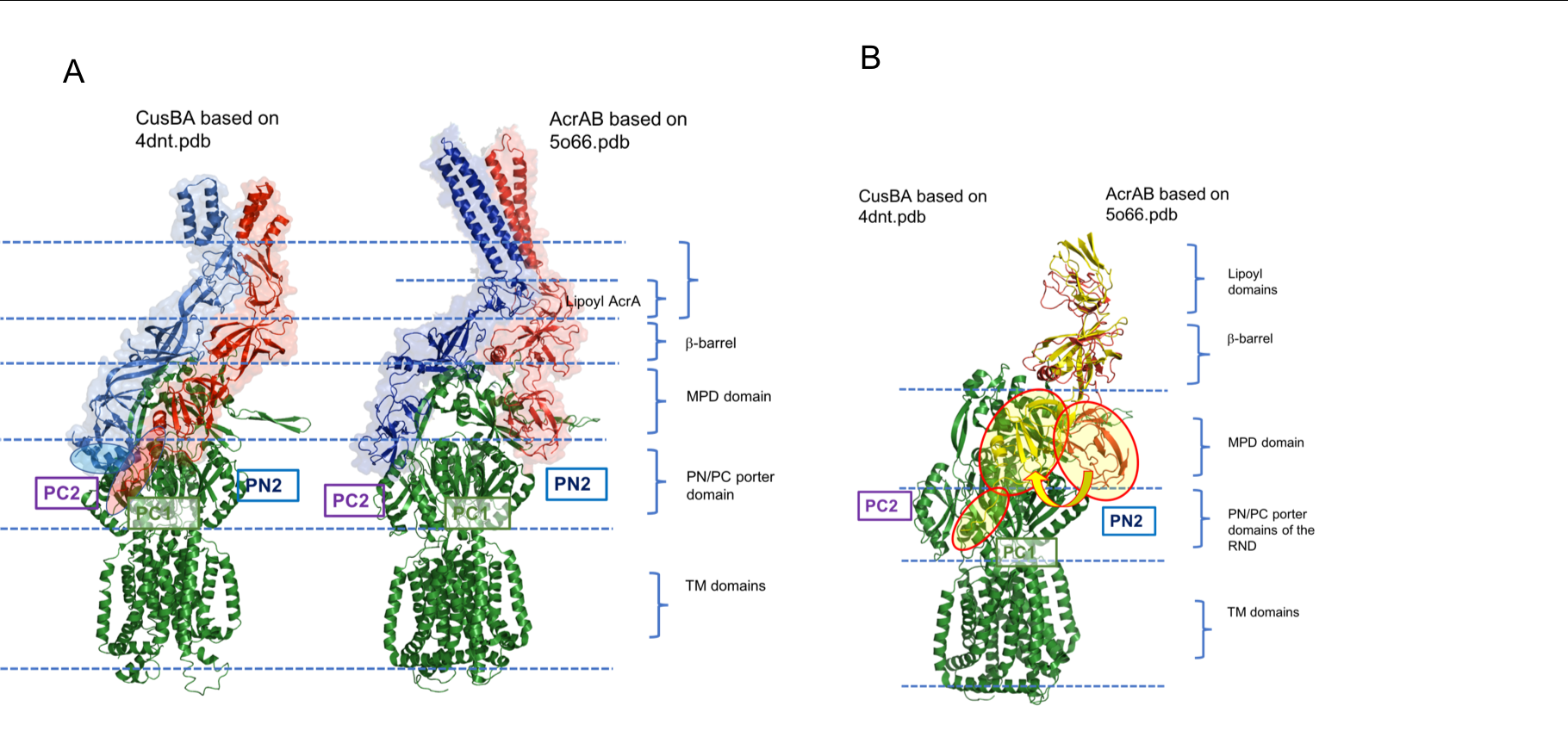

Supplement: S10 Fig — A: comparison of the PAP1 and PAP2 assembling on the CusA surface (left) and AcrB surface (right). Note the clear difference in PAP2 interaction with PC1 and PN2 domains. The lipoyl domains in CusBA complex also are much more vertically extended and present a steeper angle relative to the funnel domain of the transporter. B: Superposition of the PAP2 orientation in AcrAB complex (red) and CusBA complex–yellow. For clarity the beta-hairpin domain is removed. While beta-barrel domains are in similar orientation the linker to the MP domain seems to have undergone a large conformational change as a result of which the MP domain is interacting primarily with PC1 domain, but appears to have lost the PN2-domain interactions. Notably the N- and C-termini of the PAP2 in CusBA complex form extended contacts with the cleft between PC1/PC2 subdomains, which is homologous to the entry 2 tunnel of the drug efflux transporters. (TIF) [file ppat.1008101.s010.tif]
