# Supplementary material for: Identification of binding residues between periplasmic adapter protein (PAP) and RND efflux pumps explains PAP-pump promiscuity and roles in antimicrobial resistance
Source: PLoS Pathog. 2019 Dec 26;15(12):e1008101. doi: 10.1371/journal.ppat.1008101 (PMC6975555; doi:10.1371/journal.ppat.1008101)
Supplement: S1 Table — Data presented is the mean of at least three independent biological replicates +/- SEM. (DOCX) [file ppat.1008101.s011.docx]

Table S1. Complete Strain list with generation time and ethidium bromide efflux where measured.

| Genotype | Strain | Mean Generation Time (min) ± SEM |  | Mean time taken for fluorescence due to ethidium bromide to drop (min) | | | | |
| --- | --- | --- | --- | --- | --- | --- | --- | --- |
|  |  |  |  | 10% ± SEM |  | 20% ± SEM |  | 50% ± SEM |
| WT (SL1344) | SE01 | 29.1 ± 2.9 |  | 3.3 ± 0.4 |  | 5.4 ± 0.5 |  | 9.6 ± 1.3 |
| *ΔacrB* | SE02 | 27.9 ± 2.6 |  | 6.6 ± 0.5 |  | 12.3 ± 1.0 |  | 24.7 ± 3.0 |
| *ΔacrA* | SE03 | 29.8 ± 2.5 |  | 6.5 ± 0.5 |  | 12.3 ± 1.2 |  | 20.0 ± 1.1 |
| *ΔacrE* | SE04 | 26.5 ± 2.2 |  | 3.3 ± 0.6 |  | 5.6 ± 1.2 |  | 10.6 ± 3.1 |
| *ΔmdsA* | SE05 | 35.0 ± 5.4 |  | 3.7 ± 0.9 |  | 6.5 ± 1.6 |  | 16.1 ± 7.9 |
| *ΔmdtA* | SE06 | 24.7 ± 0.9 |  | 3.7 ± 0.6 |  | 6.0 ± 1.1 |  | 13.0 ± 4.8 |
| *ΔacrA ΔacrE* | SE07 | 27.4 ± 0.9 |  | 7.8 ± 0.4 |  | 15.4 ± 0.8 |  | 32.7 ± 3.3 |
| *ΔacrA ΔmdsA* | SE20 |  |  |  |  |  |  |  |
| *ΔacrA ΔmdtA* | SE21 |  |  |  |  |  |  |  |
| *ΔacrE ΔmdsA* | SE15 | 48.2 |  |  |  |  |  |  |
| *ΔacrE ΔmdtA* | SE16 | 23.0 |  |  |  |  |  |  |
| *ΔmdsA ΔmdtA* | SE257 |  |  |  |  |  |  |  |
| *ΔacrA ΔacrE ΔmdsA* | SE08 | 26.2 ± 1.0 |  |  |  |  |  |  |
| *ΔacrA ΔacrE ΔmdtA* | SE09 | 28.0 ± 2.1 |  |  |  |  |  |  |
| *ΔacrA ΔmdsA ΔmtsA* | SE22 |  |  |  |  |  |  |  |
| *ΔacrE ΔmdsA ΔmdtA* | SE17 | 34.5 |  |  |  |  |  |  |
| *ΔacrA ΔacrE ΔmdsA ΔmdtA* (Δ4PAP) | SE10 | 23.3 ± 2.6 |  | 10.6 ± 1.3 |  | 19.5 ± 2.0 |  | 43.0 ± 5.9 |
| *Δ4PAP + pET20b EV* | SE25 | 33.7 |  | 10.2 ± 2.6 |  | 19.4 ± 5.0 |  | 49.9 ± 20.4 |
| *Δ4PAP + pET20b acrA* | SE26 | 31.6 |  | 3.1 ± 0.1 |  | 5.4 ± 0.2 |  | 8.8 ± 0.6 |
| *Δ4PAP + pET20b acrE* | SE31 | 26.8 |  | 9.0 ± 2.4 |  | 18.8 ± 3.2 |  | 38.5 ± 10.0 |
| *Δ4PAP + pET20b mdsA* | SE33 |  |  | 6.5 ± 1.6 |  | 15.2 ± 2.2 |  | 31.4 ± 5.7 |
| *Δ4PAP + pET20b mdtA* | SE32 |  |  | 10.1 ± 1.8 |  | 17.9 ± 2.4 |  | 33.9 ± 6.5 |
| *Δ4PAP +pTrc acrA* | SE11 | 31.1 ± 4.4 |  | 12.2 ± 1.4 |  | 22.1 ± 3.1 |  | 49.3 ± 23.1 |
| *Δ4PAP +pTrc acrE* | SE12 | 30.5 ± 1.9 |  | 6.7 ± 2.1 |  | 13.0 ± 5.3 |  | 19.2 ± 9.9 |
| *Δ4PAP +pTrc mdsA* | SE13 | 31.7 ± 0.9 |  | 7.9 ± 1.0 |  | 14.8 ± 2.3 |  | 37.3 ± 11.5 |
| *Δ4PAP +pTrc ΔmdtA* | SE14 | 29.4 ± 1.0 |  | 9.0 ± 1.0 |  | 17.9 ± 1.6 |  | 47.0 ± 9.0 |
| *Δ4PAP ΔacrF* | SE141 | 27.0 |  | 7.1 ± 0.9 |  | 16.1 ± 1.7 |  | 38.1 ± 3.8 |
| *Δ4PAP ΔacrB* | SE143 | 27.1 |  | 7.4 ± 0.8 |  | 16.9 ± 1.3 |  | 36.6 ± 1.9 |
| *Δ4PAP ΔacrF +pTrc acrA* | SE168 |  |  | 1.9 ± 0.3 |  | 3.6 ± 0.5 |  | 6.0 ± 0.8 |
| *Δ4PAP ΔacrB +pTrc acrA* | SE176 |  |  | 5.7 ± 0.4 |  | 14.0 ± 0.7 |  | 32.2 ± 5.1 |
| *Δ4PAP ΔacrF +pTrc acrE* | SE169 |  |  | 4.2 ± 0.2 |  | 8.3 ± 0.4 |  | 16.4 ± 1.3 |
| *Δ4PAP ΔacrB +pTrc acrE* | SE177 |  |  | 6.3 ± 0.5 |  | 15.8 ± 0.3 |  | 35.7 ± 1.5 |
| *Δ4PAP ΔacrF+ pTrc mdsA* | SE170 |  |  |  |  |  |  |  |
| *Δ4PAP ΔacrB + pTrc mdsA* | SE178 |  |  |  |  |  |  |  |
| *Δ4PAP ΔacrF+ pTrc mdtA* | SE170 |  |  |  |  |  |  |  |
| *Δ4PAP ΔacrB + pTrc mdtA* | SE179 |  |  |  |  |  |  |  |
| *Δ4PAP ΔacrF + pET20b acrA* | SE172 | 22.3 |  |  |  |  |  |  |
| *Δ4PAP ΔacrB + pET20b acrA* | SE180 | 29.6 |  |  |  |  |  |  |
| *Δ4PAP ΔacrF + pET20b acrE* | SE173 | 28.1 |  |  |  |  |  |  |
| *Δ4PAP ΔacrB + pET20b acrE* | SE181 | 22.0 |  |  |  |  |  |  |
| *Δ4PAP ΔacrF + pET20b mdsA* | SE174 |  |  |  |  |  |  |  |
| *Δ4PAP ΔacrB + pET20b mdsA* | SE182 |  |  |  |  |  |  |  |
| *Δ4PAP ΔacrF + pET20b mdtA* | SE175 |  |  |  |  |  |  |  |
| *Δ4PAP ΔacrB + pET20b mdtA* | SE183 |  |  |  |  |  |  |  |
| *M15 (ΔacrA)* | SE186 |  |  |  |  |  |  |  |
| *M16 (ΔacrA)* | M16 |  |  |  |  |  |  |  |
| *M17 (ΔacrA)* | M17 |  |  |  |  |  |  |  |
| *M18 (ΔacrA)* | M18 |  |  |  |  |  |  |  |
| *M19 (ΔacrA)* | M19 |  |  |  |  |  |  |  |
| *M20 (ΔacrA)* | M20 |  |  |  |  |  |  |  |
| *M15 acrB::aph* | SE262 |  |  |  |  |  |  |  |
| *M15 acrF::aph* | SE263 |  |  |  |  |  |  |  |
