# Supplementary material for: Identification of binding residues between periplasmic adapter protein (PAP) and RND efflux pumps explains PAP-pump promiscuity and roles in antimicrobial resistance
Source: PLoS Pathog. 2019 Dec 26;15(12):e1008101. doi: 10.1371/journal.ppat.1008101 (PMC6975555; doi:10.1371/journal.ppat.1008101)
Supplement: S2 Table — (DOCX) [file ppat.1008101.s012.docx]

| Strains | MIC (μg/mL) | | | | | | | | | | | | |  |
| --- | --- | --- | --- | --- | --- | --- | --- | --- | --- | --- | --- | --- | --- | --- |
|  | CIP | NAL | CHL | TET | ERY | FUS | NOV | STR | ACR | CV | EtBr | MB | R6G | |
| WT | 0.032 | 4 | 4 | 1 | 128 | >1024 | 512 | 8 | 128 | 64 | >1024 | >1024 | >1024 | |
| Δ*acrB* | 0.008 | 1 | 1 | 0.5 | 4 | 8 | 4 | 8 | 16 | 4 | 64 | 16 | 16 | |
| Δ4PAP | 0.008 | 1 | 1 | 0.5 | 16 | 8 | 2 | 4 | 32 | 4 | 32 | 16 | 8 | |
| Δ4PAP p*acrA* | 0.008 | 1 | 2 | 1 | 128 | 512 | 128 | 4 | 64 | 16 | 512 | 256 | 256 | |
| Δ*acrAB* | 0.008 | 1 | 1 | 0.5 | 4 | 8 | 2 | 8 | 32 | 4 | 64 | 16 | 16 | |
| Δ4PAP p*acrA* G58F | 0.008 | 4 | 1 | 0.5 | 8 | 8 | 4 | 4 | 16 | 4 | 16 | 16 | 8 | |
| Δ4PAP p*acrA* T217F | 0.008 | 1 | 4 | 1 | 128 | 512 | 256 | 4 | 64 | 16 | 512 | 256 | 256 | |
| Δ4PAP p*acrA* S220E | 0.008 | 1 | 2 | 1 | 128 | 512 | 128 | 4 | 64 | 16 | 512 | 256 | 256 | |
| Δ4PAP p*acrA* D250F | 0.008 | 2 | 4 | 1 | 128 | 512 | 128 | 4 | 64 | 16 | 512 | 256 | 256 | |
| Δ4PAP p*acrA* TT270-271FF | 0.008 | 1 | 1 | 0.5 | 8 | 8 | 2 | 4 | 16 | 4 | 32 | 32 | 16 | |
| Δ4PAP p*acrA* GS272-273PP | 0.008 | 1 | 1 | 0.5 | 8 | 8 | 2 | 4 | 16 | 4 | 16 | 16 | 8 | |
| Δ4PAP p*acrA* F292G | 0.008 | 2 | 1 | 0.5 | 8 | 8 | 4 | 4 | 32 | 4 | 64 | 32 | 16 | |
| Δ4PAP p*acrA* R294F | 0.008 | 1 | 1 | 0.5 | 8 | 8 | 2 | 4 | 16 | 4 | 32 | 16 | 8 | |
| Δ4PAP p*acrA* Q310F | 0.008 | 1 | 2 | 1 | 128 | 512 | 64 | 4 | 64 | 16 | 512 | 256 | 256 | |
| Δ4PAP p*acrA* R315F | 0.008 | 1 | 2 | 0.5 | 128 | 512 | 128 | 4 | 64 | 16 | 512 | 256 | 256 | |
| Δ4PAP p*acrA* R318A | 0.008 | 1 | 2 | 1 | 128 | 512 | 32 | 4 | 64 | 16 | 512 | 256 | 256 | |
| Δ4PAP p*acrA* E333F | 0.008 | 1 | 2 | 1 | 128 | 512 | 128 | 4 | 64 | 16 | 512 | 256 | 256 | |
| Δ4PAP p*acrA* D345R | 0.008 | 1 | 2 | 1 | 128 | 512 | 128 | 4 | 64 | 16 | 512 | 256 | 256 | |
| Δ4PAP p*acrA* G363F | 0.008 | 1 | 1 | 0.5 | 8 | 8 | 4 | 4 | 32 | 4 | 128 | 32 | 64 | |
| Δ4PAP p*acrA* R368D | 0.008 | 1 | 2 | 1 | 128 | 512 | 128 | 4 | 64 | 16 | 512 | 256 | 256 | |

CIP, ciprofloxacin; NAL, nalidixic acid; CHL, chloramphenicol; TET, tetracycline; ERY, erythromycin; FUS, fusidic acid; NOV, novobiocin; STR, streptomycin; ACR, acriflavine; CV, crystal violet; EtBr, ethidium bromide; MB, methylene blue; R6G, rhodamine 6G
