# Supplementary material for: Identification of binding residues between periplasmic adapter protein (PAP) and RND efflux pumps explains PAP-pump promiscuity and roles in antimicrobial resistance
Source: PLoS Pathog. 2019 Dec 26;15(12):e1008101. doi: 10.1371/journal.ppat.1008101 (PMC6975555; doi:10.1371/journal.ppat.1008101)
Supplement: S1 Text — (DOCX) [file ppat.1008101.s013.docx]

**Supplementary Text.**

**Supplementary results** **text**

First we modelled the *Salmonella* AcrA (Uniprot ID Q8ZRA6_SALTY) based on the *E. coli* homologue. There is no gap in the alignment between the two with a pairwise equivalence of the sequence and identical numbering from N- to C-termini. The two sequences present 91.4% identity (34 substitutions over 397 residue overlap), with zero gaps, and could be effectively treated as point mutants of each other.

While the level of identity between the *Salmonella* AcrA and AcrE (Uniprot ID Q8ZLN5_SALTY) is significantly lower (69.3%), the alignment between their sequences reveals just a single amino-acid gap at position 39, (corresponding to the variable post-signal-sequence region), with the rest of the structure presenting *a direct pairwise correspondence*, that translates into an unequivocal assignment of secondary structure and a predicted RMSD of AcrA and AcrE is less than 0.5 Angst over full length C-alpha backbone. In particular, the membrane proximal domains were virtually identical in terms of fold and structure based on high level of identity and lack of any gaps in the structural alignment (RMSD <0.4 Å) (Figure 3A). However, while structure is conserved there is a loop between residues 327 and 331 of AcrA (326 to 330 of AcrE) that varies significantly in sequence between the two proteins. Comparing the position of this loop to the recent cryoEM structure of Wang and colleagues suggests that this loop does not contact with AcrB but is surface exposed (1).

In contrast, the sequences, and correspondingly the predicted structures of both MdtA (Uniprot ID Q8ZNQ3 (MDTA_SALTY)) and MdsA (Q8ZRG8_SALTY) are markedly different with identities below 30% and significant gaps in the alignments. The models suggested that MdtA and MdsA were most similar to each other (RMSD <0.3 Å) but much less similar to AcrA and AcrE both in terms of overall structure and specifically the structure of membrane proximal domain (RMSD 1-2 Å). Both have a significantly more extended C-termini bringing the overall length to 413 residues in the case of MdtA), and more hydrophobic N-termini, as well as a clear shortening of the alpha helix 4 (see Espript structural alignment presented in Figure 3a ). In addition, MdsA has also an extended loop between beta10 and beta11; while MdtA also features an N-terminal insertion (residues 37-54), of unknown function, which could possibly correspond to a transmembrane anchoring domain.

Despite the above differences, which are mostly restricted to the C- and N-termini, and a couple of variable loops, the core of the structure and in particular the hairpin tips which are presumed to interact with the OMF TolC are well conserved – notably the ‘RLSD” signature sequence is present in all of them (2). The overall structure of the beta-barrel and MP domains is also conserved across the family (Figure S2).

**Supplementary discussion text**

Our structural analysis has also provided several additional insights regarding the PAP-RND interaction. The AcrB trimer has several intramolecular solvent accessible channels that have been hypothesised to play the role of drug-extrusion conduits (3-5). 3 main conduits have been identified leading to a main binding pocket, each with their separate entry. The first (entrance 1) is within the inner membrane (6), the second (entrance 2) at above membrane level in the intraprotomer cleft (PC1/PC2 interface) (7), as well as the central-cavity access channel (5). These entrances all lead to a proximal drug-binding pocket in “access” (“loose”) protomer, and the cargo is then transferred to the deeper distal binding pocket in the “binding” (“tight”) protomer. Proximal and distal binding pockets are separated by a switch loop (containing F617) and in “extrusion” (“open”) protomer a new, outward conduit is created towards the AcrA/TolC funnel, while the entry pockets are closed, allowing the substrate to diffuse out of the cell. In addition, there is evidence that the inter-protomer clefts, also referred to as a “vestibules” may function as substrate entry pathways (8). It is remarkable that both PAP1 and PAP2 associate closely with the PN2/PC1 lobe of the periplasmic domain of AcrB, and thus both protomers are flanking the primary entrance (entrance 2) to the proximal binding pocket, while the entrance 1 and the vestibule pathway (located between the protomers) are away from any visible interaction. As these alternative entry pathways have been shown to also bind substrate (8) it is thus tempting to suggest, that perhaps PAPs contribute to the selectivity and processivity of the pump *via* entrance 2 for a sub-group of substrates, while substrates are pumped *via* entry 1 and vestibules in a non-PAP dependent fashion.

Our analysis of the association of PAP2 with PN2 observed in the cryo-EM structures provides a further hint at functional speciation between the PAP protomers. Early experiments with chimeric transporters formed of part AcrB and part MexB, (9) have shown that the specificity of PAP-RND association is restricted within the residue range 60-612, which covers the first structural repeat of the RND protein (from PN1, PN2, to TM7 and major part of PC1 domain). The new structural data suggests that PAP2, and not PAP1 is involved in this selection, which also suggests that the PAP2 is likely to participate in substrate presentation. The functioning of the chimeric assemblies which are roughly 50:50 AcrB/MexB also suggests, that PAP1 plays more of a generic role in stabilizing the assembly, and may be promiscuous (Figure S9).

While all the current cryo-EM derived structures, including those obtained in the presence of substrate, appear to be symmetric and present similar conformations of PAP1 and PAP2 relative to the RND protomers, it needs to be stressed that PAPs might be able to undergo significant conformational changes during the efflux cycle which are not necessarily reflected in the currently available artificially stabilized pump complexes. Indeed the analysis of the currently only available crystal structures of PAP-RND co-complex - namely CusBA (10) including the only asymmetric structure of the complex - 4DNT.pdb (11) demonstrates a PAP2 conformation and interaction with RND-transporter which strongly deviates from the cryo-EM complexes discussed (Figure S10).

Whether this is down to the specialized nature of the CusBA as a metal transporter is hard to say, but considering that the CusB structure in the complex with CusA transporter is the only one to show defined the extended C- and N-termini of the PAP, and these appear to form extensive contacts with the PC1/PC2 cleft which is the primary site of drug entry in the efflux transporters such as AcrB (corresponding to entry tunnel No2 discussed above) it is possible that this conformation reflects on some intermediate functional transport state.

Finally, our analysis of the site-directed mutations introduced into the sequence boxes and their subsequent mapping onto the structure of the assembled complexes revealed some unanticipated parallels between the previously described asymmetric structures of AcrB (7, 12) and the PAP-engaged cryo-EM structures (1, 13). Namely, the detectable effect of the Q310F mutation is quite unexpected, given that Q310 is predicted to only make contact with the RND transporter in PAP protomer 1. A closer inspection however, revealed that in asymmetric cryo-EM structures Q310 appears to coordinate the AcrB funnel domain hairpin (Nβ8-Nβ9 hairpin) from the neighbouring RND protomer, which is implicated in the oligomerisation of the RND-protomers. It is notable that in the asymmetric DARPin-stabilised structures of the AcrB (e.g. 4u8v.pdb (12)) one of the DARPin molecules interacts with the same region of the Nβ8-Nβ9 hairpin as the AcrA Q310. It is tempting to suggest that such interaction mimics the functional interaction provided by the PAP and can be used by the PAP for either sensing of the substrate occupation state of the neighboring RND protomer possibly stabilizing a L-to-T transition, or for active coupling of the conformational states of the RND protomers during the cycle. While future research is needed to elucidate the exact role of these mutations, our data narrows down dramatically the range of residues involved and provides a testable hypothesis for the role of PAPs in the assembly and conformational communication during the efflux cycle.

1. Wang Z, Fan G, Hryc CF, Blaza JN, Serysheva, II, Schmid MF, Chiu W, Luisi BF, Du D. 2017. An allosteric transport mechanism for the AcrAB-TolC multidrug efflux pump. Elife 6.

2. Lee M, Jun SY, Yoon BY, Song S, Lee K, Ha NC. 2012. Membrane fusion proteins of type I secretion system and tripartite efflux pumps share a binding motif for TolC in gram-negative bacteria. PLoS One 7:e40460.

3. Yamaguchi A, Nakashima R, Sakurai K. 2015. Structural basis of RND-type multidrug exporters. Front Microbiol 6:327.

4. Nakashima R, Sakurai K, Yamasaki S, Hayashi K, Nagata C, Hoshino K, Onodera Y, Nishino K, Yamaguchi A. 2013. Structural basis for the inhibition of bacterial multidrug exporters. Nature 500:102-6.

5. Nakashima R, Sakurai K, Yamasaki S, Nishino K, Yamaguchi A. 2011. Structures of the multidrug exporter AcrB reveal a proximal multisite drug-binding pocket. Nature 480:565-9.

6. Murakami S, Nakashima R, Yamashita E, Matsumoto T, Yamaguchi A. 2006. Crystal structures of a multidrug transporter reveal a functionally rotating mechanism. Nature 443:173-179.

7. Seeger MA, Schiefner A, Eicher T, Verrey F, Diederichs K, Pos KM. 2006. Structural asymmetry of AcrB trimer suggests a peristaltic pump mechanism. Science 313:1295-8.

8. Husain F, Bikhchandani M, Nikaido H. 2011. Vestibules are part of the substrate path in the multidrug efflux transporter AcrB of *Escherichia coli*. J Bacteriol 193:5847-9.

9. Tikhonova EB, Wang Q, Zgurskaya HI. 2002. Chimeric analysis of the multicomponent multidrug efflux transporters from gram-negative bacteria. J Bacteriol 184:6499-507.

10. Su CC, Long F, Zimmermann MT, Rajashankar KR, Jernigan RL, Yu EW. 2011. Crystal structure of the CusBA heavy-metal efflux complex of *Escherichia coli*. Nature 470:558-62.

11. Su CC, Long F, Lei HT, Bolla JR, Do SV, Rajashankar KR, Yu EW. 2012. Charged amino acids (R83, E567, D617, E625, R669, and K678) of CusA are required for metal ion transport in the Cus efflux system. J Mol Biol 422:429-41.

12. Eicher T, Seeger MA, Anselmi C, Zhou W, Brandstatter L, Verrey F, Diederichs K, Faraldo-Gomez JD, Pos KM. 2014. Coupling of remote alternating-access transport mechanisms for protons and substrates in the multidrug efflux pump AcrB. Elife 3.

13. Tsutsumi K, Yonehara R, Ishizaka-Ikeda E, Miyazaki N, Maeda S, Iwasaki K, Nakagawa A, Yamashita E. 2019. Structures of the wild-type MexAB-OprM tripartite pump reveal its complex formation and drug efflux mechanism. Nat Commun 10:1520.
